# Associations between pulmonary function and depression: evidence from the CHARLS cohort 2015–2018

**DOI:** 10.3389/fpubh.2025.1551356

**Published:** 2025-07-17

**Authors:** Hui Yang, Juan Cao, Lin Huang, Zhijun Xie, Yuejun Zhou, Yiqing Wang, Chengping Wen

**Affiliations:** ^1^College of Basic Medical Sciences, Zhejiang Chinese Medical University, Hangzhou, China; ^2^Medical College of Zhejiang University, Hangzhou, China; ^3^College of Basic Medical Sciences, Shanghai University of Traditional Chinese Medicine, Shanghai, China

**Keywords:** depression, peak expiratory flow, CHARLS, prospective cohort study, cognitive

## Abstract

**Background:**

The relationship between lung function and depression in middle-aged and older adults remains poorly understood. To address this knowledge gap, we conducted cross-sectional and longitudinal analyses using data from the nationally representative China Health and Retirement Longitudinal Study (CHARLS) to examine the association between peak expiratory flow (PEF) status and depression among middle-aged and older Chinese adults.

**Methods:**

A total of 15,137 participants aged 45 years and older were included from the 2015 China Health and Retirement Longitudinal Study (CHARLS). Participants were categorized into three PEF status groups based on their percentage of predicted PEF: severe impairment (<80%), average lung function (80–100%), and good lung function (≥100%). A total of 12,304 participants were included in the longitudinal analysis from 2015 to 2018. To address potential confounding, propensity score matching was employed using a gradient-boosting model to balance covariates between participants with and without depression. Logistic regression analyses as well as restricted cubic spline (RCS) regression analyses were performed to examine the association between (PEF) status and depression incidence.

**Findings:**

The prevalence of depression was 9.53% (*n* = 1,044) in the cross-sectional sample, with higher rates observed in participants with poorer lung function (9.53, 7.11, and 5.03% for good, moderately good, and poorer lung function, respectively). During a 3.6-year follow-up, 6.73% (*n* = 130) of participants developed depression. Fully adjusted logistic regression analysis demonstrated a significant inverse linear association between PEF percentage and depression risk (OR: 0.898, 95% CI: 0.862–0.935, per 1 SD increase). These findings were corroborated by restricted cubic spline (RCS) regression analysis, which revealed a linear relationship without evidence of nonlinearity (*P* for nonlinearity = 0.631).

**Conclusion:**

Our study revealed a noteworthy correlation between PEF percentage and depression among the middle-aged and older population. The PEF percentage emerges as a valuable tool that may enhance the primary prevention and treatment of depression.

## Introduction

Depression is a common mental disorder worldwide and a leading cause of disability (1). Mental health problems, especially depression, among the old people, represent a significant public health challenge in aging societies (2). Moreover, the prevalence of depression tends to increase with age (3, 4). In China, where the old people population is projected to reach 400 million by 2050 (5–7), depression affects over 95 million individuals nationwide, making it a major mental health concern (8).

Depression may be a complex outcome, particularly in the onset process among old people individuals. A multivariate logistic regression analysis showed that depressive symptoms in old people patients are associated with education level, financial status, exercise habits, occurrence of chronic diseases, and loneliness (9). Social isolation in particular is a significant issue among the old people population in today’s world. A cohort study involving 25,482 people in Japan found that the prevalence of social isolation increased from 21 to 28% during the COVID-19 pandemic (10).

Previous studies have found that the occurrence of depression in the old people may be associated with major risk factors for late-life depression including gender, rural residence, living alone, low monthly income, unhealthy lifestyle, major medical conditions, and psychosocial problems. The incidence of depressive disorders is significantly higher in women than in men, and higher in rural areas than in urban areas. This includes disparities between rural and urban areas in terms of economic levels, social services, and healthcare access (11–13).

Systematic review evidence has confirmed the antidepressant effects of exercise on late-life depression (14, 15), while the association between lung function and the occurrence of depression remains unclear. A comprehensive review of 1,161,632 individuals showed that moderate to high pulmonary fitness correlates with reduced depression risk (16). Supporting this finding, a longitudinal study of middle-aged and older Chinese adults demonstrated that increased depressive symptoms significantly correlated with reduced PEF (*b* = −1.85, *p* < 0.001), with men experiencing a notably steeper decline (b = −2.36, *p* < 0.001) compared to women (*b* = −1.46, *p* < 0.001) during periods of heightened depressive symptoms (17). These findings suggest that better psychological health could help minimize the impact of reduced pulmonary function and decrease depression risk. Depression often occurs alongside cognitive impairments, indicating that disrupted interactions in cognition-related brain networks may underlie its varied symptoms (18).

Using data from the China Health and Retirement Longitudinal Study (CHARLS), we examined the relationship between lung function and depression in middle-aged and older Chinese adults.

While previous studies have examined the relationship between depressive symptoms and lung function, their findings have been inconsistent (19, 20).

Furthermore, unlike Western countries, the treatment rate for depression among old people in China is extremely low, which may be related to the high level of stigma that old people attach to depression, which is tied to Chinese social and cultural factors (21). For example, in a sample of urban Chinese primary care old people patients, the treatment rate for major depression was less than 1% (22). This indicates that there are substantial unmet mental health needs among old people Chinese patients. Analysis of the CHARLS cohort helps examine the overall situation of depression among China’s old people population.

Therefore, our study aims to address this knowledge gap by using a larger sample size to investigate the effects of depressive symptoms on lung function in Chinese adults without pulmonary diseases. Through both cross-sectional and longitudinal analyses, we investigated how impaired lung function contributes to depression prevalence. Our findings indicate that peak expiratory flow (PEF), an easily measurable indicator of lung function, could serve as a useful biomarker for early depression detection.

## Methods

### Data and study participants

CHARLS is a nationally representative longitudinal study following Chinese adults aged 45 and older since its inception in 2011. The study captures detailed information on participants’ health, socioeconomic conditions, and lifestyle through regular in-person interviews and standardized questionnaires.

A nationally representative sample of 17,708 individuals from 10,257 households across China’s 28 provinces was selected using a multi-stage stratified probability proportional sampling method. Participants have been followed up every 2 years since the study began. The data incorporates weighting variables to maintain national representativeness. Detailed information about the study design of the CHARLS has been previously reported (23).

This study retrospectively analyzed data from the 2015 and 2018 waves of the CHARLS survey. Inclusion criteria were: (1) being aged 45 or older in the 2015 CHARLS survey; and (2) having complete data on peak expiratory flow rate. Exclusion criteria were: (1) missing data on peak expiratory flow rate in the 2015 CHARLS survey; (2) missing age information; (3) being younger than 45 years old; (4) lacking essential data from the cognitive and depression assessments; and (5) pre-existing diagnosis of respiratory diseases.

A two-part study was conducted. The cross-sectional analysis involved a retrospective review of data from the 2015 wave of the CHARLS. Of the 21,801 participants, 5,958 were excluded due to missing PEF data (*n* = 6,257), age less than 45 years (*n* = 325), or missing data on key cognitive and depression variables (*n* = 31). The final analytic sample for the cross-sectional analysis consisted of 15,188 participants.

**Figure 1:**
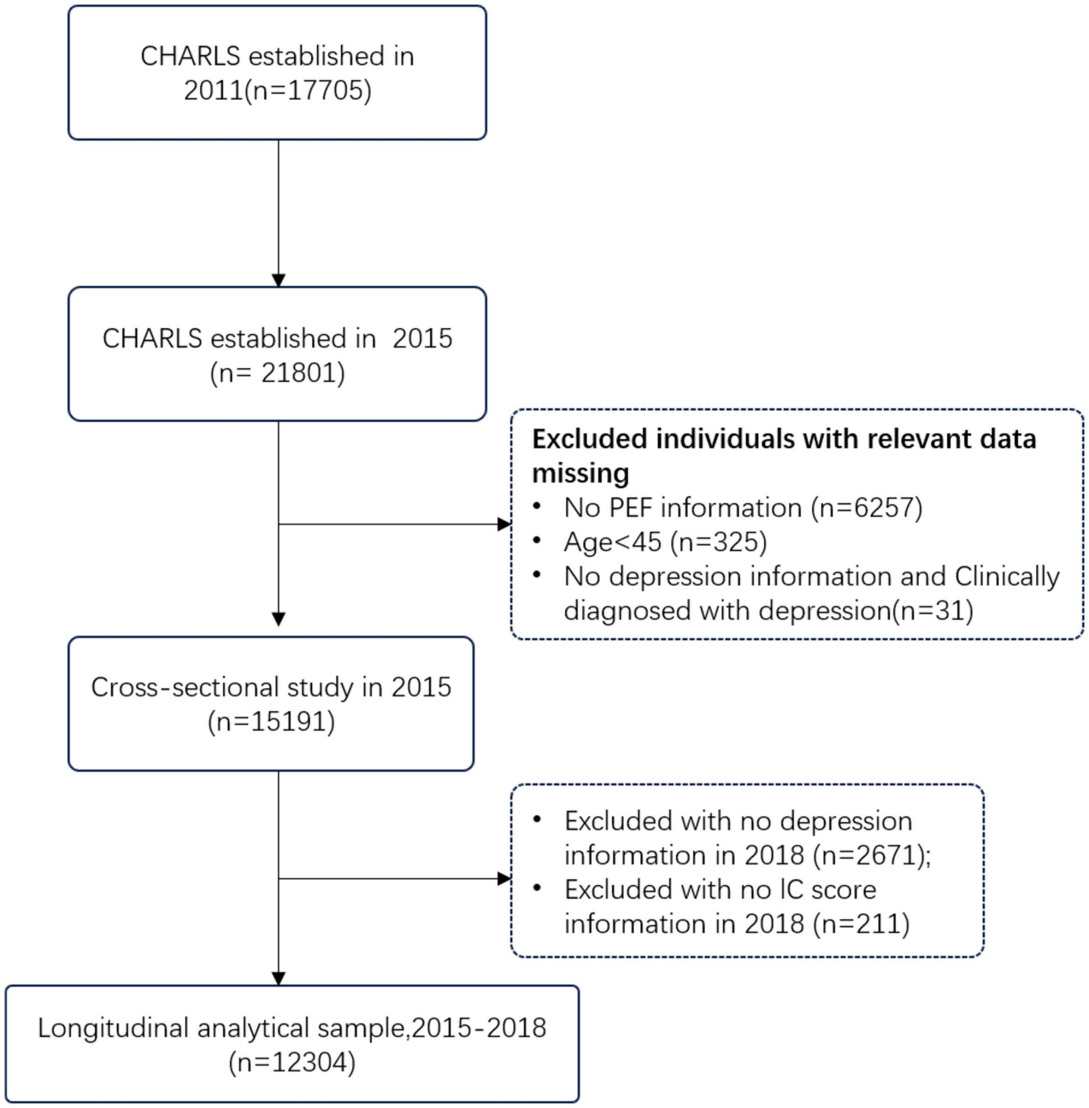
Study cohort. Inclusion and exclusion criteria for the study cohort (*n* = 12,304).

For the longitudinal analysis, a subset of the CHARLS cohort was selected. Participants with a diagnosis of depression in 2015 were excluded. After excluding those lost to follow-up in 2018 (*n* = 2,671) and those without IC scores in 2018 (*n* = 211), the final analytic sample for the longitudinal analysis consisted of 12,304 participants. The selection process is depicted in Figure 1.

This study adhered to the Declaration of Helsinki and relevant ethical standards. All participants provided written informed consent after receiving detailed information about the study. The study protocol was approved by the Institutional Review Board of Peking University (IRB00001052-11,015). To ensure methodological rigor, this study strictly followed the STROBE guidelines.

### Interview procedures and quality control

The CHARLS survey employs a rigorous and standardized questionnaire administration process to ensure data quality. Trained interviewers (undergraduate/graduate students) conducted in-person, computer-assisted personal interviews (CAPI) using standardized questionnaires. Interviewers underwent rigorous training, including mock interviews and examinations, to ensure protocol adherence. For participants unable to respond (e.g., due to cognitive impairment), proxy informants provided answers. Supervisors monitored fieldwork to resolve technical issues and ensure data accuracy. The CAPI system enabled real-time error detection and correction. Detailed methodology and quality control measures are described elsewhere (24).

### Measurement of peak expiratory flow

Peak expiratory flow (PEF) was measured using a portable peak flow meter (Everpure™, Shanghai, China) and a disposable mouthpiece. Trained technicians obtained three PEF measurements per participant, using the highest value for analysis. Participants were instructed to stand upright, inhale deeply, and exhale forcefully into the mouthpiece. A 30-s rest period was observed between measurements.

To account for the unique characteristics of the Chinese population, we employed the peak expiratory flow (PEF) prediction equations proposed by Zhong Nanshan et al (25). Based on the Chinese national census data. These equations apply to adults aged 18–80 years and are expressed as follows: for males, PEF (L/min) = 75.6 + 20.4 × A - 0.41 × A^2^ + 0.002 × A^3^ + 1.19 × H; for females, PEF (L/min) = 282.0 + 1.79 × A - 0.046 × A^2^ + 0.68 × H, where A represents age and H represents height.

Our main analyses used a widely accepted fixed cutoff point of 80% of predicted PEF, with scores less than 80% of predicted PEF indicating low PEF values (26). We classified participants into three groups based on their percentage of predicted PEF: severe impairment (<80%), Average lung function (80–100%), and good lung function (≥100%). These categories were used to characterize the status of airflow obstruction.

### Assessment of depression

Depressive symptoms were assessed at baseline using the Chinese version of the Center for Epidemiologic Studies Depression Scale (CES-D-10), a validated instrument for measuring depression in Chinese old people populations (27, 28). The CES-D-10 consists of 10 items assessing depressive symptoms experienced in the past week, with two positively worded items. The total score ranged from 0 to 30, with higher scores indicating more severe depressive symptoms.

### Assessment of sarcopenia

Sarcopenia was assessed using the AWGS 2019 algorithm, incorporating muscle strength, appendicular skeletal muscle mass (ASM), and physical performance. Grip strength was measured in both hands using the Yuejian TM WL-1000 dynamometer (Nantong Yuejian Physical Fitness Measuring Instrument Co., Ltd., Nantong, China). ASM was estimated using validated anthropometric equations specific to the Chinese population. Low muscle mass was defined as an ASM/height^2^ value below 5.63 kg/m^2^ for women and 7.05 kg/m^2^ for men.

### Assessment of intrinsic capacity score

An intrinsic capacity (IC) score was computed, encompassing physical capacity (1 km walk completion), vitality (self-rated health), sensory function (vision and hearing), psychological well-being (depressive symptoms), and cognitive function (word memory, calculation, episodic memory, drawing). The IC score was structured into an overall domain and five subdomains (physical, cognitive, vitality, sensory, psychological), for each subdomain, a binary score was assigned: 1 if there was evidence of impairment and 0 if there was no impairment. The total IC score was calculated by summing the scores across all five subdomains.

### Covariates

Baseline sociodemographic data (age, gender, education, marital status, residence) and health-related information were collected through structured interviews conducted by trained interviewers. To comprehensively explore the multifaceted etiology of depression, a Gradient Boosting Machine (GBM) model was employed. A wide range of covariates, including individual-level biological, psychological, and social factors (e.g., marital status, childhood adversities, socioeconomic indicators), were incorporated into the GBM model to examine their intricate relationships with depression. Although the amount of missing data was relatively small, multiple imputation was performed using the mice package in R to address potential biases. The algorithm was configured with m = 5 imputations, balancing statistical efficiency and computational feasibility, and maxit = 20 iterations to ensure convergence (verified via trace plots). Predictive mean matching (PMM) was applied for continuous variables, while binary variables were imputed using logistic regression, with variable-specific methods explicitly defined in the meth argument. A detailed overview of the missing data pattern is presented in Supplementary Figure S1.

### Statistical analysis

A gradient boosting model (GBM) was employed to estimate propensity scores for depression, mitigating covariate imbalance between depression and non-depression groups. GBM iteratively constructs new models, forming an ensemble that optimizes a loss function. We chose to use the Gradient Boosting Machine (GBM) method because it’s good at finding complex patterns in data without needing detailed instructions. This is especially useful for studying depression because depression has many different causes - from physical and mental health factors to social and economic conditions, as well as early life experiences. Other methods that Random Forests, while capable of handling complex relationships, offer less precision than GBM; Support Vector Machines (SVM) perform well but present challenges in setup and scalability with large datasets; and logistic regression, though more interpretable, is less effective at analyzing complex variable interactions.

Baseline characteristics of cross-sectional and longitudinal study participants were summarized according to the percentage of predicted peak expiratory flow rate. Categorical variables were compared using Chi-square or Fisher’s exact tests, as appropriate. Continuous variables were compared using one-way ANOVA followed by Tukey’s *post hoc* test if normally distributed, or the Kruskal-Wallis test otherwise. Continuous variables are presented as mean (SD), and categorical variables as percentages.

Cross-sectional associations between lung function status (potential impairment, average, good) and depression were assessed using logistic regression analysis. Additionally, a longitudinal analysis was conducted to estimate the incidence rate of depression per 1,000 person-years using the 2018 wave of the CHARLS dataset. The relationship between baseline lung function and incident depression was evaluated using relative risks (RRs) and 95% confidence intervals (CIs), with subsequent subgroup analyses.

To assess the impact of demographic, health, and socioeconomic factors on the outcome, three models were estimated in both cross-sectional and longitudinal analyses. Model 1 adjusted for gender and age. Model 2 was further adjusted for marital status, self-reported health status, and childhood health. Model 3 included all variables from Model 2, plus satisfaction with marriage, ASMHt^2^, satisfaction with local services, physical impairments (eyesight and hearing), economic factors (pension insurance), self-rated memory, sleep duration, difficulty with stooping, kneeling, and crouching, sleeping time, systolic blood pressure, and pulse.

The statistical analyses were performed using SPSS version 25.0 (SPSS, Inc., Chicago, IL) and R version 4.3.2 (R Foundation for Statistical Computing) for the retrospective analysis. A *p*-value of less than 0.05 was considered statistically significant in all cases.

## Result

### Characteristics of participants in the cross-sectional and longitudinal studies

Baseline characteristics of the study population are presented in Table 1. A total of 15,188 participants were included, with a mean age of 68.53 (SD 10.08) years.

**Table 1:** Baseline characteristics of all participants stratified by ratio of peak expiratory flow rate.

Covariates	Level	Overall	Restricted	Average	Good	*p*
n		15,188	4,449	3,837	6,902	
Demographic and lifestyle						
Age (mean (SD))		68.53 (10.08)	68.50 (10.53)	66.56 (9.62)	69.64 (9.87)	<0.001
Gender (%)	Male	7,115 (46.85)	1891 (42.50)	1,668 (43.47)	3,556 (51.52)	<0.001
	Female	8,073 (53.15)	2,558 (57.50)	2,169 (56.53)	3,346 (48.48)	
Arm length (cm) (mean (SD))		33.56 (11.39)	33.41 (14.64)	33.54 (15.83)	33.67 (2.88)	0.495
Knee Height (cm) (mean (SD))		47.82 (13.85)	47.42 (14.71)	47.51 (3.65)	48.25 (16.58)	0.002
Location (%)	1 Main City Zone	1901 (12.56)	460 (10.37)	438 (11.45)	1,003 (14.58)	<0.001
	2 Combination Zone Between Urban and Rural Areas	707 (4.67)	180 (4.06)	205 (5.36)	322 (4.68)	
	3 The Town Center	804 (5.31)	216 (4.87)	202 (5.28)	386 (5.61)	
	4 ZhenXiang Area	407 (2.69)	115 (2.59)	107 (2.80)	185 (2.69)	
	5 Special Area	57 (0.38)	15 (0.34)	12 (0.31)	30 (0.44)	
	6 Township Central	211 (1.39)	73 (1.65)	45 (1.18)	93 (1.35)	
	7 Village	11,052 (73.00)	3,375 (76.12)	2,816 (73.62)	4,861 (70.65)	
Marital status (%)	1 Married with Spouse Present	12,617 (83.07)	3,615 (81.25)	3,228 (84.13)	5,774 (83.66)	<0.001
	2 Married But Not Living with Spouse Temporarily for Reasons Such as Work	725 (4.77)	227 (5.10)	201 (5.24)	297 (4.30)	
	3 Separated	33 (0.22)	12 (0.27)	10 (0.26)	11 (0.16)	
	4 Divorced	123 (0.81)	42 (0.94)	26 (0.68)	55 (0.80)	
	5 Widowed	1,581 (10.41)	510 (11.46)	344 (8.97)	727 (10.53)	
	6 Never Married	93 (0.61)	41 (0.92)	22 (0.57)	30 (0.43)	
	7 Cohabitated	16 (0.11)	2 (0.04)	6 (0.16)	8 (0.12)	
Smoking status (%)	1 Still Have	4,246 (65.05)	1,225 (64.61)	1,085 (70.50)	1936 (62.61)	<0.001
	2 Quit	1917 (29.37)	573 (30.22)	374 (24.30)	970 (31.37)	
	3 Never Smoked	364 (5.58)	98 (5.17)	80 (5.20)	186 (6.02)	
Alcohol use (%)	1 I Never Had A Drink	8,536 (76.63)	2,692 (78.76)	2,230 (78.36)	3,614 (74.13)	<0.001
	2 I Used to Drink Less Than Once A Month	1,282 (11.51)	332 (9.71)	308 (10.82)	642 (13.17)	
	3 I Used to Drink More Than Once A Month	1,321 (11.86)	394 (11.53)	308 (10.82)	619 (12.70)	
Days Ag work (mean (SD))		5.24 (2.08)	5.33 (2.03)	5.26 (2.04)	5.15 (2.14)	0.007
Ed change (%)	1 No Formal Education (Illiterate)	2,887 (26.43)	945 (30.11)	690 (26.34)	1,252 (24.24)	<0.001
	2 Did Not Finish Primary School But Capable of Reading/Writing	2040 (18.68)	667 (21.26)	484 (18.47)	889 (17.22)	
	3 Sichu/Home School	40 (0.37)	13 (0.41)	14 (0.53)	13 (0.25)	
	4 Elementary School	2,520 (23.07)	748 (23.84)	590 (22.52)	1,182 (22.89)	
	5 Middle School	2,292 (20.99)	553 (17.62)	593 (22.63)	1,146 (22.19)	
	6 High School	772 (7.07)	144 (4.59)	188 (7.18)	440 (8.52)	
	7 Vocational School	212 (1.94)	47 (1.50)	42 (1.60)	123 (2.38)	
	8 Two/Three Year College/Associate Degree	113 (1.03)	14 (0.45)	13 (0.50)	86 (1.67)	
	9 Four-year College/Bachelor’s Degree	45 (0.41)	7 (0.22)	6 (0.23)	32 (0.62)	
	10 Master’s Degree	1 (0.01)	0 (0.00)	0 (0.00)	1 (0.02)	
Marriage sat (%)	1 Completely Satisfied	1,001 (7.55)	252 (6.62)	263 (7.71)	486 (8.04)	<0.001
	2 Very Satisfied	5,574 (42.02)	1,554 (40.82)	1,403 (41.11)	2,617 (43.29)	
	3 Some What Satisfied	5,657 (42.65)	1,662 (43.66)	1,456 (42.66)	2,539 (42.00)	
	4 Not Very Satisfied	723 (5.45)	235 (6.17)	203 (5.95)	285 (4.71)	
	5 Not at All Satisfied	305 (2.30)	102 (2.68)	88 (2.58)	115 (1.90)	
	6 No Spouse Now	5 (0.04)	2 (0.05)	0 (0.00)	3 (0.05)	
Mos Ag work (mean (SD))		6.40 (4.17)	6.68 (4.17)	6.36 (4.16)	6.23 (4.18)	<0.001
Childhood Health (%)	1 Excellent	1738 (11.59)	440 (10.03)	431 (11.38)	867 (12.70)	<0.001
	2 Very Good	6,068 (40.46)	1,617 (36.88)	1,521 (40.17)	2,930 (42.93)	
	3 Good	2,847 (18.99)	911 (20.78)	720 (19.02)	1,216 (17.82)	
	4 Fair	3,372 (22.49)	1,085 (24.74)	877 (23.16)	1,410 (20.66)	
	5 Poor	971 (6.48)	332 (7.57)	237 (6.26)	402 (5.89)	
Midday sleep time (hrs) (mean (SD))		38.75 (44.95)	38.55 (45.55)	37.25 (44.94)	39.71 (44.55)	0.024
Sleep time (hrs) (mean (SD))		6.40 (1.92)	6.37 (2.03)	6.41 (1.92)	6.42 (1.85)	0.333
Self-reported health						
Health sat (%)	1 Completely Satisfied	544 (3.61)	140 (3.18)	144 (3.78)	260 (3.79)	<0.001
	2 Very Satisfied	3,190 (21.16)	894 (20.30)	777 (20.39)	1,519 (22.15)	
	3 Some What Satisfied	7,442 (49.37)	2038 (46.27)	1865 (48.94)	3,539 (51.60)	
	4 Not Very Satisfied	2,937 (19.48)	960 (21.79)	769 (20.18)	1,208 (17.61)	
	5 Not at All Satisfied	961 (6.38)	373 (8.47)	256 (6.72)	332 (4.84)	
Toilet diff (%)	1 No, I Do not Have Any Difficulty	8,239 (81.57)	2,623 (80.14)	2040 (81.89)	3,576 (82.47)	0.044
	2 I Have Difficulty But Can Still Do It	1,564 (15.49)	532 (16.25)	390 (15.66)	642 (14.81)	
	3 Yes, I Have Difficulty and Need Help	167 (1.65)	71 (2.17)	33 (1.32)	63 (1.45)	
	4 I Can Not Do It	130 (1.29)	47 (1.44)	28 (1.12)	55 (1.27)	
Life sat (%)	1 Completely Satisfied	995 (6.59)	281 (6.37)	236 (6.18)	478 (6.95)	0.001
	2 Very Satisfied	5,510 (36.49)	1,608 (36.48)	1,362 (35.69)	2,540 (36.95)	
	3 Some What Satisfied	7,326 (48.52)	2091 (47.44)	1880 (49.27)	3,355 (48.81)	
	4 Not Very Satisfied	1,015 (6.72)	335 (7.60)	273 (7.15)	407 (5.92)	
	5 Not at All Satisfied	252 (1.67)	93 (2.11)	65 (1.70)	94 (1.37)	
Self-rated memory (%)	1 Excellent	127 (0.84)	31 (0.70)	34 (0.89)	62 (0.90)	<0.001
	2 Very good	835 (5.50)	228 (5.13)	211 (5.50)	396 (5.74)	
	3 Good	1,142 (7.53)	307 (6.91)	290 (7.57)	545 (7.90)	
	4 Fair	8,118 (53.51)	2,264 (50.97)	2021 (52.73)	3,833 (55.58)	
	5 Poor	4,949 (32.62)	1,612 (36.29)	1,277 (33.32)	2060 (29.87)	
Sitting diff (%)	1 No, I Do not Have Any Difficulty	10,532 (71.63)	2,943 (68.11)	2,643 (72.04)	4,946 (73.68)	<0.001
	2 I Have Difficulty But Can Still Do It	3,842 (26.13)	1,241 (28.72)	952 (25.95)	1,649 (24.56)	
	3 Yes, I Have Difficulty and Need Help	210 (1.43)	84 (1.94)	54 (1.47)	72 (1.07)	
	4 I Can Not Do It	119 (0.81)	53 (1.23)	20 (0.55)	46 (0.69)	
Stooping diff (%)	1 No, I Do not Have Any Difficulty	9,945 (67.70)	2,719 (62.98)	2,513 (68.53)	4,713 (70.29)	<0.001
	2 I Have Difficulty But Can Still Do It	3,208 (21.84)	997 (23.09)	778 (21.22)	1,433 (21.37)	
	3 Yes, I Have Difficulty and Need Help	270 (1.84)	104 (2.41)	73 (1.99)	93 (1.39)	
	4 I Can Not Do It	1,266 (8.62)	497 (11.51)	303 (8.26)	466 (6.95)	
Difficulty with climbing (%)	1 No, I Do not Have Any Difficulty	8,563 (58.79)	2,185 (51.18)	2,150 (59.24)	4,228 (63.42)	<0.001
	2 I Have Difficulty But Can Still Do It	4,021 (27.61)	1,262 (29.56)	1,004 (27.67)	1755 (26.32)	
	3 Yes, I Have Difficulty and Need Help	348 (2.39)	133 (3.12)	84 (2.31)	131 (1.96)	
	4 I Can Not Do It	1,633 (11.21)	689 (16.14)	391 (10.77)	553 (8.29)	
Health change (%)	1 Better	1,442 (9.68)	467 (10.71)	342 (9.11)	633 (9.33)	<0.001
	2 About The Same	7,120 (47.79)	1886 (43.25)	1820 (48.47)	3,414 (50.34)	
	3 Worse	6,336 (42.53)	2008 (46.04)	1,593 (42.42)	2,735 (40.33)	
Disease aware (%)	1 Yes	105 (0.71)	35 (0.81)	29 (0.77)	41 (0.61)	<0.001
	2 No	12,960 (87.18)	3,657 (84.40)	3,281 (87.24)	6,022 (88.92)	
	3 Do not Know	1801 (12.11)	641 (14.79)	451 (11.99)	709 (10.47)	
Self-rated health (%)	1 Excellent	117 (1.52)	26 (1.15)	33 (1.70)	58 (1.67)	<0.001
	2 Very Good	947 (12.32)	224 (9.88)	237 (12.20)	486 (13.98)	
	3 Good	955 (12.43)	239 (10.54)	235 (12.09)	481 (13.84)	
	4 Fair	4,349 (56.58)	1,267 (55.89)	1,105 (56.87)	1977 (56.88)	
	5 Poor	1,318 (17.15)	511 (22.54)	333 (17.14)	474 (13.64)	
Ill in Last month (%)	1 Yes	1814 (14.30)	609 (16.78)	473 (14.78)	732 (12.51)	<0.001
	2 No	10,867 (85.70)	3,020 (83.22)	2,727 (85.22)	5,120 (87.49)	
Disability (%)	1 Yes	2,181 (14.38)	812 (18.30)	547 (14.27)	822 (11.92)	<0.001
	2 No	12,014 (79.20)	3,283 (73.97)	3,089 (80.59)	5,642 (81.79)	
	3 Too Old to Do The Work	974 (6.42)	343 (7.73)	197 (5.14)	434 (6.29)	
Lifting Diff (%)	1 No, I Do not Have Any Difficulty	12,794 (87.16)	3,528 (81.82)	3,190 (87.13)	6,076 (90.62)	<0.001
	2 I Have Difficulty But Can Still Do It	744 (5.07)	267 (6.19)	215 (5.87)	262 (3.91)	
	3 Yes, I Have Difficulty and Need Help	145 (0.99)	55 (1.28)	40 (1.09)	50 (0.75)	
	4 I Can Not Do It	995 (6.78)	462 (10.71)	216 (5.90)	317 (4.73)	
Local service sat (%)	1 Very Satisfied	2,299 (15.64)	722 (16.78)	582 (15.62)	995 (14.92)	0.023
	2 Somewhat Satisfied	3,564 (24.25)	1,041 (24.20)	870 (23.35)	1,653 (24.78)	
	3 Neutral	6,001 (40.83)	1758 (40.86)	1,528 (41.01)	2,715 (40.70)	
	4 Somewhat disSatisfied	1809 (12.31)	472 (10.97)	479 (12.86)	858 (12.86)	
	5 Very disSatisfied	1,025 (6.97)	309 (7.18)	267 (7.17)	449 (6.73)	
Run/Jog 1 Km (%)	1 No, I Do not Have Any Difficulty	7,011 (48.89)	1,675 (39.82)	1778 (49.79)	3,558 (54.20)	<0.001
	2 I Have Difficulty But Can Still Do It	1,587 (11.07)	440 (10.46)	417 (11.68)	730 (11.12)	
	3 Yes, I Have Difficulty and Need Help	189 (1.32)	63 (1.50)	51 (1.43)	75 (1.14)	
	4 I Can Not Do It	5,554 (38.73)	2028 (48.22)	1,325 (37.10)	2,201 (33.53)	
Vision status (mean (SD))		0.69 (0.46)	0.72 (0.45)	0.70 (0.46)	0.68 (0.47)	<0.001
Auditory status (mean (SD))		0.65 (0.48)	0.67 (0.47)	0.65 (0.48)	0.63 (0.48)	<0.001
Label index						
Systolic BP, mmHg (mean (SD))		129.19 (46.45)	128.41 (44.58)	127.96 (42.22)	130.37 (49.73)	0.016
Pulse, bpm (mean (SD))		74.20 (10.83)	75.01 (11.35)	74.05 (10.61)	73.76 (10.57)	<0.001
Platelets, x10^9/L (mean (SD))		204.93 (74.73)	207.13 (75.32)	203.06 (73.00)	204.58 (75.27)	0.079
Diastolic BP, mmHg (mean (SD))		75.19 (12.14)	74.76 (12.57)	75.32 (12.16)	75.39 (11.84)	0.02
maxPEF, L/min (mean (SD))		321.66 (125.39)	198.57 (72.28)	309.06 (71.93)	408.02 (105.07)	<0.001
HDL Chol, mg/dL (mean (SD))		51.22 (11.50)	51.77 (11.65)	51.19 (11.47)	50.90 (11.42)	0.002
Hemoglobin, g/dL (mean (SD))		13.72 (1.95)	13.60 (1.97)	13.68 (1.99)	13.81 (1.90)	<0.001
CRP, mg/L(mean (SD))		2.65 (5.63)	2.96 (6.07)	2.63 (5.77)	2.48 (5.24)	<0.001
WBC, x10^9/L(mean (SD))		5.99 (2.07)	6.03 (1.88)	5.96 (2.67)	5.99 (1.79)	0.393
HbA1c, (%) (mean (SD))		5.97 (0.98)	5.95 (0.94)	5.93 (0.97)	6.00 (1.02)	0.002
Total Chol, mg/dL (mean (SD))		183.91 (36.37)	181.70 (36.86)	183.60 (36.17)	185.46 (36.10)	<0.001
Economic indicators						
New rural pension (%)	1 Participate	4,672 (54.95)	1,436 (54.44)	1,373 (62.21)	1863 (50.93)	<0.001
	2 Receive	3,831 (45.05)	1,202 (45.56)	834 (37.79)	1795 (49.07)	
Hrs Ag work (mean (SD))		6.45 (2.99)	6.62 (2.92)	6.50 (2.97)	6.30 (3.04)	0.001
Pension Insur (%)	1 Yes, Have Qualification	8,214 (56.43)	2,464 (58.04)	2,129 (57.79)	3,621 (54.63)	<0.001
	2 Yes, Other Reason	727 (4.99)	227 (5.35)	187 (5.08)	313 (4.72)	
	3 No, Participate in Government or Firm Pension	1,520 (10.44)	300 (7.07)	318 (8.63)	902 (13.61)	
	4 No, Local Community Not Execute	1952 (13.41)	576 (13.57)	502 (13.63)	874 (13.19)	
	5 No, Other Reason	2,144 (14.73)	678 (15.97)	548 (14.88)	918 (13.85)	
Composite indicators						
TyG_BMI (mean (SD))		32.65 (3.91)	32.17 (4.10)	32.62 (3.90)	32.97 (3.77)	<0.001
ASM/Ht^2 score (mean (SD))		11.83 (3.14)	11.12 (3.22)	11.83 (3.10)	12.29 (3.01)	<0.001
Sarcopenia (%)	No	14,167 (94.57)	3,984 (91.10)	3,590 (94.87)	6,593 (96.61)	<0.001
	Yes	814 (5.43)	389 (8.90)	194 (5.13)	231 (3.39)	
rec_score (mean (SD))		13.12 (5.77)	11.97 (5.83)	13.17 (5.72)	13.83 (5.65)	<0.001
IC_score (mean (SD))		4.27 (1.38)	4.49 (1.35)	4.27 (1.37)	4.13 (1.38)	<0.001
rec_score_level (%)	Good	2,765 (18.21)	577 (12.97)	690 (17.98)	1,498 (21.70)	<0.001
	Bad	12,423 (81.79)	3,872 (87.03)	3,147 (82.02)	5,404 (78.30)	
PEF percentage (mean (SD))		96.52 (31.88)	60.10 (15.85)	90.22 (5.66)	123.51 (21.24)	<0.001
Depression (%)	Normal	9,921 (65.32)	2,619 (58.87)	2,487 (64.82)	4,815 (69.76)	<0.001
	Mild	2,638 (17.37)	839 (18.86)	675 (17.59)	1,124 (16.29)	
	Moderate	1,585 (10.44)	567 (12.74)	402 (10.48)	616 (8.92)	
	Severe	1,044 (6.87)	424 (9.53)	273 (7.11)	347 (5.03)	

*p < 0.05, **p < 0.01, and ***p < 0.001 indicate statistical significance.

Participants were categorized into three groups based on their peak expiratory flow (PEF) percentage: restricted, average, and good. The restricted, average, and good groups comprised 4,449, 3,837, and 6,902 participants, respectively. Depression prevalence was significantly higher in the restricted group 424 (9.53) compared to the average 273 (7.11) and good 347 (5.03) groups (*p* < 0.001).

Compared to the restricted group, participants in the good group were older (69.64 vs. 66.56 years, *p* < 0.001), had a higher proportion of males (51.52% vs. 48.48%, *p* < 0.001), lower sarcopenia prevalence (3.39% vs. 8.90%, *p* < 0.001), higher physical activity levels (rec score: 13.83 vs. 11.97, *p* < 0.001), and better body composition, higher ASM/Ht^2^ score and lower TyG BMI.

The IC score, a composite measure of overall health, was significantly lower in the good group (mean IC score ± SD: 4.13 ± 1.38) compared to the restricted (4.49 ± 1.35) and average (4.27 ± 1.38) groups (*p* < 0.001). Typically, higher IC scores indicate better overall health.

Cross-Sectional Associations of Potential Airflow Limitation with Depression and Cognition.

**Figure 2:**
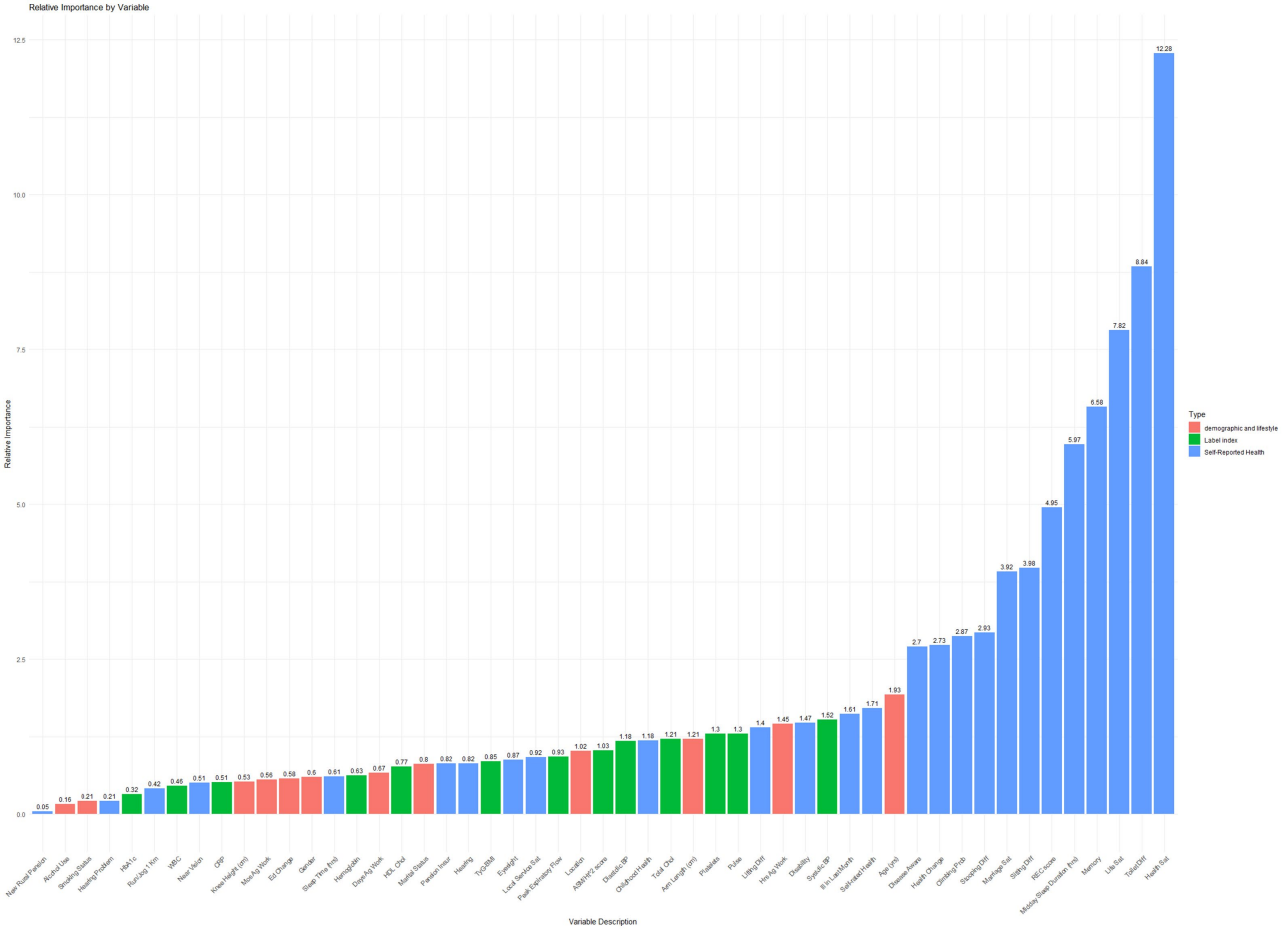
Illustrates the relative importance of these covariates in predicting propensity scores.

A Gradient Boosting Machine (GBM) model was developed using regression trees as base learners and trained on a dataset of 15,188 observations and 53 covariates. The model demonstrated strong discriminative power in predicting depression, achieving an AUC of 0.8438. To assess model stability, 3-fold cross-validation, and early stopping were implemented. Additional performance metrics included accuracy (0.784), precision (0.803), recall (0.888), and F1-score (0.843). The relative importance of covariates in predicting propensity scores is illustrated in Figure 2.

Key factors influencing propensity scores were self-reported health, functional limitations (e.g., difficulty with toileting, and sitting), and self-reported health-related quality of life (including life satisfaction, health satisfaction, and sleep quality, encompassing both nighttime and midday sleep).

Notably, physical health factors, including respiratory function (peak expiratory flow, relative importance: 0.931) and muscle mass (ASM/Ht^2^, relative importance: 1.030), were strongly associated with propensity scores. Socioeconomic factors, such as the demanding nature of household agricultural work (relative importance: 1.453) and marital satisfaction (relative importance: 3.919), significantly influenced propensity scores. Additionally, childhood health (relative importance: 1.184) emerged as a crucial determinant.

These findings underscore the complex interplay of factors influencing propensity scores for depression in older adults. A comprehensive approach addressing physical, psychological, social, economic, and early life factors is essential for effective prevention and management strategies.

### Association between the PEF percentages and depression incidence

The prevalence of depression in the cross-sectional sample was 9.53% (1,044/15,188) overall, with rates of 9.53% (424/4,449), 7.11% (273/3,837), and 5.03% (347/6,902) observed in participants with good, moderately good, and poorer lung function, respectively (Table 2). Fully adjusted logistic regression models demonstrated an inverse association between higher levels of PEF percentage and depression risk. Compared to those with lower PEF percentages, individuals with higher levels had lower odds of depression (OR = 0.812, 95% CI: 0.740–0.891, respectively). Moreover, when treating PEF percentage as a continuous variable, a one standard deviation increase in PEF percentage was associated with lower odds of depression (OR = 0.898, 95% CI: 0.862–0.935).

**Table 2:** Stratified analysis of PEF percentage and depression stratified by different levels of PEF in baseline.

PEF Percentage	Restricted	Average	Good	*p* value	P for trend	Per 1 SD increase
Median(PEF Percentage)	60.10 (15.85)	90.22 (5.66)	123.51 (21.24)		-	-
Cases, n (%)	4,449	3,837	6,902		-	-
Crude, OR (95% CI)	Reference	0.777(0.711–0.849)	0.620(0.573–0.671)	<0.001	< 0.001	0.802(0.775–0.83)
Model 1, OR (95% CI)	Reference	0.804(0.734–0.880)	0.635(0.586–0.688)	<0.001	< 0.001	0.804(0.777–0.833)
Model 2, OR (95% CI)	Reference	0.845(0.767–0.931)	0.714(0.656–0.778)	0.006	< 0.001	0.846(0.815–0.878)
Model 3, OR (95% CI)	Reference	0.864(0.779–0.959)	0.812(0.740–0.891)	<0.001	< 0.001	0.898(0.862–0.935)

### Longitudinal associations between baseline PEF and depression/cognitive dysfunction

Table 3 presents the incidence rates of depression and cognitive dysfunction from 2015 to 2018, stratified by baseline PEF (peak expiratory flow) percentage status (restricted, average, good). The table shows the number of new cases, the incidence rate per 1,000 individuals, and relative ratios (RR) with 95% CI from three different models (Model 1^a^, Model 2^b^, Model 3^c^). The reference group for PEF percentage is ‘Restricted’. Statistical significance is denoted by *** (*p* < 0.001).

**Table 3:** Depression incidence rates from 2015 to 2018 stratified by baseline PEF percentage status.

PEF_Percentage	New cases of depression (%n)	Incidence rate, per 1,000	Model 1^**a**^	Model 2^**b**^	Model 3^**c**^
Depression					
Restricted	90	7.60	Reference	Reference	Reference
Average	86	8.05	0.817 (0.740–0.902)***	0.839 (0.757–0.930)***	0.888 (0.789–1.000)
Good	130	6.73	0.633 (0.579–0.690)***	0.681 (0.622–0.746)***	0.776 (0.698–0.864)***
Cognitive dysfunction					
Restricted	257	21.71	Reference	Reference	Reference
Average	326	30.51	0.900 (0.790–1.026)	0.892 (0.781–1.018)	0.773 (0.672–0.890)***
Good	781	40.44	0.788 (0.702–0.885)***	0.796 (0.707–0.895)***	0.633 (0.558–0.717)***

Model 1: Adjusted for gender and age. Model 2: Further adjusted for gender, age, marital status, self-reported health status, and childhood health. Model 3: Built upon Model 2 by additionally adjusting for satisfaction with marriage, ASM/Ht^2^, satisfaction with local services, physical impairments (eyesight and hearing), economic factors (pension insurance), self-rated memory, sleep duration, difficulty with stooping, kneeling, and crouching, sleeping time, systolic blood pressure, and pulse. PEF, Percentage of Predicted Peak Expiratory Flow. *p < 0.05, **p < 0.01, and ***p < 0.001 indicate statistical significance.

As shown in the table, the incidence rate of depression was the lowest in the good PEF group (6.73 per 1,000) and highest in the restricted PEF group (7.60 per 1,000). The incidence rate of cognitive dysfunction was highest in the good PEF group (40.44 per 1,000) and lowest in the restricted PEF group (21.71 per 1,000). While a linear association between PEF and depression was observed, the relationship between PEF and cognitive dysfunction was more complex, exhibiting a non-linear pattern.

Compared to the restricted group, individuals in the average and good PEF groups had significantly lower risks of depression across all three models (Model 1^a^, Model 2^b^, and Model 3^c^; *p* < 0.001), with estimated risk reductions ranging from 11 to 37%.

To evaluate the stability of our findings, we conducted sensitivity analyses across three progressively adjusted logistic regression models using unimputed data. The results demonstrated remarkable consistency in the inverse association between pulmonary function (PEF groups) and depression risk, even without imputation for missing values. The related analysis is shown in Supplementary Table S7.

Restricted PEF demonstrated the strongest inverse association with depression across models (ORs: 0.63–0.81). Good PEF showed protective effects (ORs: 0.80–0.87), though weaker than Restricted PEF. Marital dissatisfaction levels showed a clear dose–response relationship with risk.

In all three models, both Average and Good PEF Groups consistently showed lower odds of depression compared to the Restricted PEF Group. These associations persisted after adjusting for multiple covariates, strengthening our confidence in the findings.

The analysis of unimputed data showed clear relationships between PEF groups and depression. Despite limitations from missing data, the consistent results across adjustment levels supported our conclusions. The fully adjusted model, which included health-related and socioeconomic factors, highlighted both the depression risk’s complexity and the protective role of better pulmonary function.

**Figure 3:**
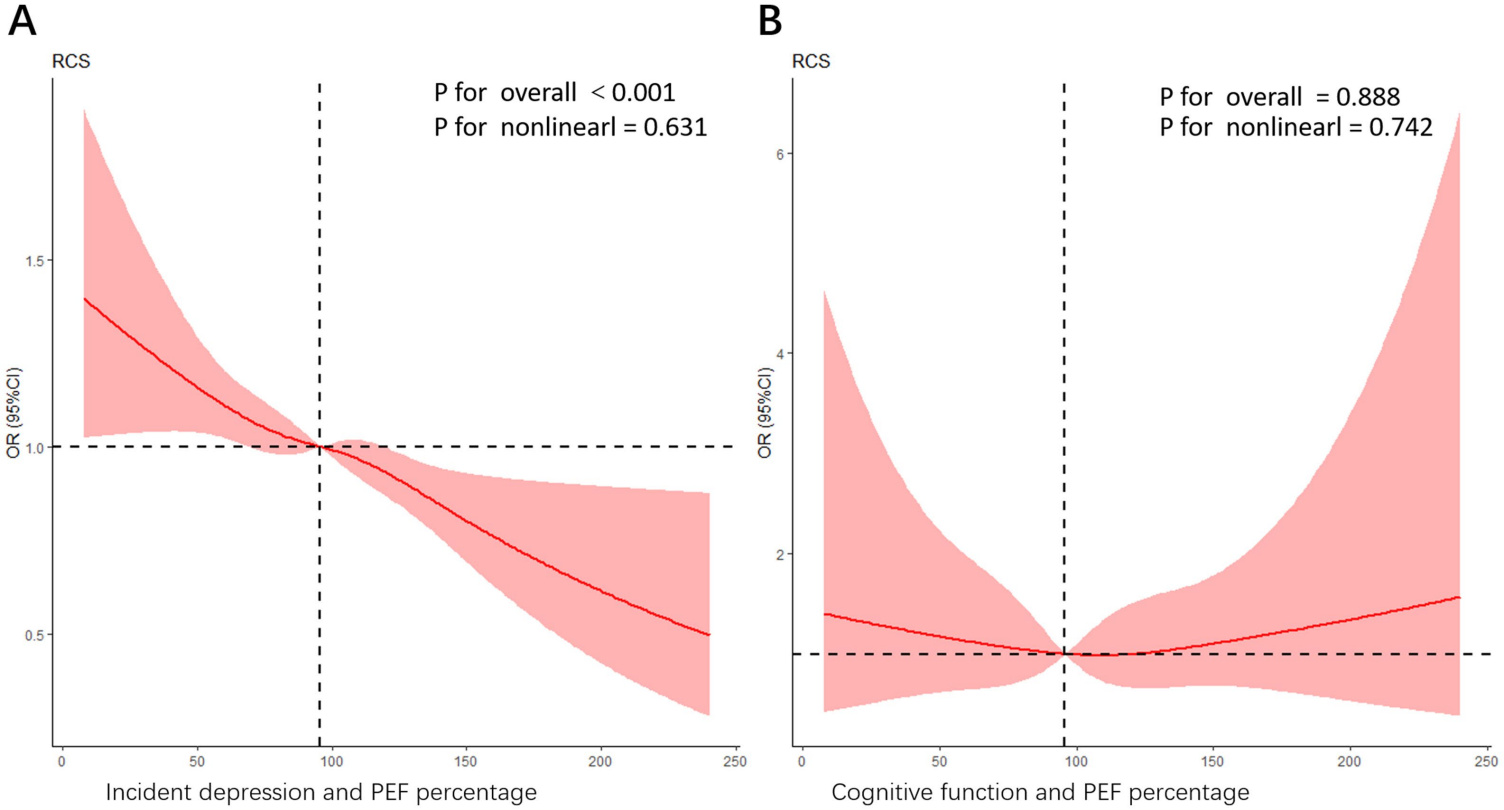
**(A)** An RCS curve illustrating the association between PEF percentage and incident depression. The model was adjusted for potential confounders including age, gender, baseline depression status, health satisfaction, childhood health, marriage satisfaction, ASM/Ht^2^, systolic blood pressure, pulse rate, eyesight, hearing, local service satisfaction, pension insurance, memory function, sleep duration (hours), difficulty with stooping and sitting difficulties. The overall significance level for the association is highly significant (*p* < 0.001), while the nonlinear trend is not significant (*P* for nonlinearity = 0.631). **(B)** An RCS curve depicting the relationship between cognitive function and PEF percentage. In this model, the overall *p*-value is 0.888, indicating that the association is not statistically significant, while the nonlinear trend *p*-value is 0.742, suggesting some nonlinearity in the relationship. The curve demonstrates a complex pattern of ORs as the PEF percentage changes. However, further interpretation should be made in light of the study design and other statistical analyses.

Figures 3A,B present restricted cubic spline (RCS) analyses assessing the association between peak expiratory flow (PEF) percentage and incident depression (Figure 3A) and cognitive function (Figure 3B), respectively. After adjusting for potential confounders, a significant nonlinear association was observed between PEF percentage and incident depression (*p* < 0.001), while no significant association was found between PEF percentage and cognitive function (*p* = 0.888). In Figure 3A, the trend reveals that in the lower range of PEF percentages, the risk of depression decreases noticeably as PEF values rise. This suggests that even modest improvements in lung function among those with poorer lung function could yield significant mental health benefits. The curve displays a steadily declining trend, emphasizing the importance of maintaining good lung function, particularly in preventing depression. In Figure 3B, Overall Significance Level: The relationship between PEF percentage and cognitive dysfunction is not statistically significant (*p* = 0.888), and the non-linear trend is also not significant (*P* for nonlinearity = 0.742). However, the curve depicts a nuanced pattern of odds ratios (ORs) as the PEF percentage varies. In the lower PEF percentage range, there seems to be a rise in the risk of cognitive dysfunction, though this trend does not achieve statistical significance. As the PEF percentage increases, the curve shows fluctuations, but no consistent trend emerges in the higher PEF percentage range to suggest that improved lung function significantly lowers the risk of cognitive dysfunction. This pattern implies that other factors—such as social isolation, nutritional status, and physical activity levels—may also play pivotal roles in the development of cognitive dysfunction.

**Figure 4:**
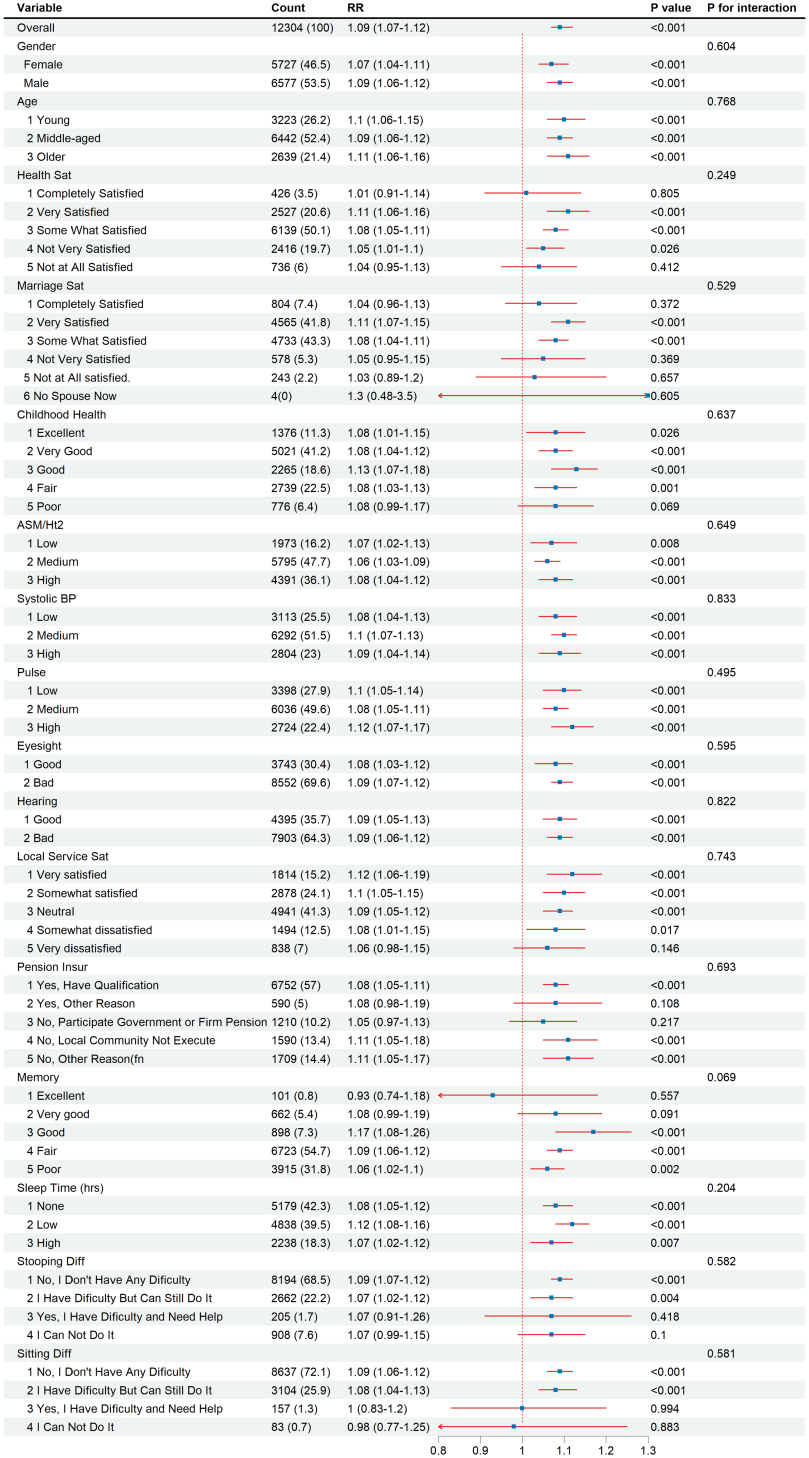
Subgroup analysis of depression risk by demographic, socioeconomic, and health-related factors, 2015–2018.

Subgroup analyses were conducted to examine factors associated with depression (Figure 4). Depression prevalence varied significantly across demographic, socioeconomic, and health-related characteristics. Individuals with lower socioeconomic status, characterized by lower education and income, exhibited elevated odds of depression. Additionally, those with poorer physical health, including chronic diseases and disabilities, demonstrated increased risk. Psychosocial factors such as limited social support, stressful life events, and adverse childhood experiences were associated with higher depression prevalence. Women, older adults, and individuals with impaired vision or hearing were also at increased risk. These findings highlight the complex interplay of multiple factors contributing to depression in this population.

## Discussion

This study utilized extensive longitudinal data from CHARLS, a nationally representative cohort, to examine the relationship between peak expiratory flow (PEF) and depression. Through controlling for multiple potential confounders and performing comprehensive subgroup analyses, we established evidence linking impaired lung function to depression.

We employed a Gradient Boosting Machine (GBM) to analyze depression risk factors (29). GBM proved ideal for handling confounding variables and precisely estimating each variable’s contribution to model predictions. In our study, we employed the Random Forest methodology for initial variable screening. Additionally, we have attached documentation describing our Random Forest screening methodology. This comprehensive approach ensures a rigorous and well-documented variable selection process. We considered a broad range of predictors across psychological, socioeconomic, relational, and physical domains, ultimately incorporating 53 variables in our analyses. Our findings support existing literature that emphasizes self-worth’s vital role in mental health (30, 31), People with higher self-esteem typically develop better coping mechanisms, maintain stronger social support networks, and exercise greater life control—all helping prevent depression. Additionally, early childhood experiences significantly influence self-concept and emotional regulation, affecting later depression vulnerability (32–35). Our study highlights how intimate relationships protect against depression, as quality marriages offer love, support, and companionship that buffer against stress and adversity (25, 36, 37). These insights suggest approaches to depression prevention and treatment by focusing on building self-esteem, fostering healthy attachment patterns, and strengthening social bonds.

Our cross-sectional analysis showed a strong correlation between the model’s predicted depression probability and actual depression prevalence, aligning with prior research (38, 39). Longitudinal analyses further revealed that old people individuals with respiratory impairment had a heightened risk of developing depression and cognitive decline, supporting previous findings (40–42).

Subgroup analyses revealed that depression risk was significantly influenced by multiple factors. Demographic variables (gender, age), socioeconomic conditions (income, education), and health-related factors (physical health status, chronic conditions) all played crucial roles in determining depression susceptibility (43, 44).

Women, older adults, and those with lower socioeconomic status or adverse psychosocial experiences demonstrated particularly elevated risks (45, 46), these comprehensive findings emphasize that depression prevention and treatment strategies must adopt an integrated approach that addresses multiple risk factors simultaneously.

Our findings demonstrate a significant association between pulmonary function and depression risk. A one standard deviation increase in PEF percentage was associated with lower odds of depression (OR = 0.898, 95% CI: 0.862–0.935, *p* < 0.001). Furthermore, individuals with lower PEF levels (restricted) showed a significantly higher risk of depression compared to those with higher PEF levels (good), with incidence rates of 7.60 and 6.73 per 1,000 (Table 3), respectively. This association remained robust after adjusting for potential confounders. These results support the novel concept of “pulmonary dysfunction-related depression,” suggesting that impaired lung function may contribute to depression development.

Participants were divided into seven regions based on where they lived: (1) Main City Zone, (2) Urban–Rural Combination Zone, (3) Town Center, (4) ZhenXiang Area, (5) Special Area, (6) Township Central, and (7) Village. There were big differences in how people were distributed across these areas (*p* < 0.001). Villages had the most participants (73.00%), while the Main City Zone had the fewest (12.56%).

These regional differences may affect the results because living conditions, medical care, and social support vary between urban and rural areas (47). For example, people in rural areas might have a higher risk of depression because they face more air pollution and worse living conditions. In contrast, people in cities might deal with more stress from work and competition, even though they have better access to healthcare.

Smoking, which harms the lungs directly and causes inflammation, was also linked to lower lung function (PEF) and higher depression risk. Current smokers had worse lung function and higher depression risk compared to former smokers and non-smokers, who had better lung health and lower depression rates (48).

Individuals with restricted cognitive function showed a significantly higher incidence rate of depression compared to those with good cognitive function (21.71 vs. 40.44 per 1,000). This association remained statistically significant after adjusting for covariates in all three models (Model 1^a^: OR = 0.773, 95% CI: 0.672–0.890, *p* < 0.001; Model 2^b^: OR = 0.796, 95% CI: 0.707–0.895, *p* < 0.001; Model 3^c^: OR = 0.633, 95% CI: 0.558–0.717, *p* < 0.001).

A complex relationship emerged between cognitive function and pulmonary function, as shown by the Restricted Cubic Spline (RCS) curve in Figure 3B. While the overall linear association between PEF percentage and cognitive function was not statistically significant (*p =* 0.880), the nonlinear trend (*p =* 0.731) indicated a possible non-linear relationship.

Regarding the inverse relationship between PEF and depression and the non-linear trend of cognitive dysfunction observed in our longitudinal analysis (2015–2018), we hypothesize that unmeasured factors such as social isolation, medication adherence, and nutritional status may contribute to this divergence. Social isolation is consistently linked to cognitive decline, particularly in older adults (49). Otherwise, medications with anticholinergic properties are commonly prescribed to older adults. The cumulative anticholinergic effect of all the medications a person takes is referred to as the anticholinergic burden, because of its potential to cause adverse effects (50). In humans, chronic malnutrition is associated with global cognitive decline and accelerated brain aging (51). However, changes in nutritional status during chronic illness may lead to cognitive decline, which could be associated with various factors such as the progression rate of the disease, cognitive state, and dietary patterns (52). This heterogeneity could contribute to non-linear patterns in our data.

Our first-wave data showed that 4,246 individuals (65.05%) were smokers. Among these participants, we found a significant correlation between poorer lung function and higher rates of severe depression. Though smoking rates were high across all lung function groups, those with moderate or poor lung function had notably higher rates of severe depression (9.53 and 7.11%, respectively) compared to those with good lung function (5.03%).

People with poorer lung function typically experience more severe chronic inflammation, leading to systemic inflammation—a known risk factor for depression (53). The oxidative stress from smoking, which worsens in those with poor lung function, can damage both lung tissue and the nervous system, increasing depression risk (54, 55). Poor cardiopulmonary function also limits physical activity and reduces quality of life, potentially causing feelings of helplessness that contribute to depression (56, 57). The higher rates of severe depression among smokers with poor lung function likely stem from multiple factors: chronic inflammation, oxidative stress, limited physical capacity, lack of social support, and medication side effects (58, 59). Our GBM model therefore included various factors beyond smoking status. While smoking was identified as an important contributor to depression, other factors such as marital status and socioeconomic conditions also played significant roles.

Prior studies have consistently linked low PEF with a higher risk of depression, though the mechanisms remain complex (60). Neuroimmune pathways, such as systemic inflammation—evidenced by elevated interleukin-6 (IL-6) and tumor necrosis factor-alpha (TNF-*α*) levels in respiratory limitations patients—may play a key role by disrupting hippocampal neurogenesis and impairing prefrontal cortex function (61, 62). Furthermore, age-related declines in lung function may amplify these effects through oxidative stress and dysregulation of brain-derived neurotrophic factor (BDNF), especially of BDNF/TrkB signaling (63). These findings underscore the importance of integrated strategies—such as combining pulmonary rehabilitation with anti-inflammatory therapies—to address both physiological and mental health outcomes.

Emerging evidence identifies the gut-lung-brain axis as a significant amplifier of the low PEF-depression connection. Chronic lung dysfunction, such as in chronic obstructive pulmonary disease (COPD), disrupts gut microbiota, leading to increased intestinal permeability and systemic inflammation. This inflammation is marked by elevated levels of pro-inflammatory cytokines like IL-6 and TNF-α (64). These cytokines can cross the blood–brain barrier, activating microglia and impairing hippocampal neuroplasticity—a process worsened by lipopolysaccharide (LPS) from dysbiotic gut bacteria (65). This axis-driven mechanism suggests that gut-targeted interventions, including probiotics or dietary adjustments, could complement pulmonary therapies to mitigate depression risk in vulnerable populations (66).

Our study has several limitations. First, we used PEF measurements to assess lung function rather than FEV1, the gold standard. This choice was driven by practical constraints in conducting large-scale epidemiological surveys in rural China. PEF devices are portable and easy to use, making them suitable for resource-limited settings and enabling broader testing coverage. Second, depression diagnoses may vary across populations, and our study focused specifically on an Asian population. Third, we lacked detailed biomarker data, which limited our exploration of underlying biological mechanisms. Fourth, due to incomplete physical examination data from the fourth wave, we could not analyze associations between depression and additional blood biomarkers, including relevant neurotransmitters like serotonin, and the longitudinal follow-up was relatively short (3.6 years). Otherwise, we used the CES-D-10 scale, a reliable tool for measuring depressive symptoms. However, self-reported data can introduce bias, as participants may underreport or over report symptoms due to social pressure or memory errors. Cultural differences might also influence how individuals interpret and respond to the questions(despite varying levels of cultural background, the cognitive scores that need to be achieved differ), and regional disparities in living conditions and healthcare access might have confounded the results, though the study adjusted for some socioeconomic factors (67). Future studies should consider exploring these regional disparities in more detail to better understand their potential impact on the relationship between lung function and depression. In our study, 2,671 participants dropped out during the follow-up period, potentially introducing bias. Those who dropped out may have been at higher risk of depression or faced other unmeasured health challenges. Future research should also prioritize diverse populations to enhance the reliability of findings, strengthen the link between PEF and mental health, and identify group-specific factors that may require tailored interventions.

Future studies should use different ways to measure mental health, like talking to patients directly, testing blood samples, and doing thorough mental health checks. This would give us a better understanding of how mental health problems develop. Despite these limitations, our findings offer valuable insights into the relationship between respiratory limitations and depression, highlighting the need for further research to understand the underlying mechanisms and develop targeted interventions. Our study demonstrates that lung function assessment could serve as a useful tool for identifying individuals at risk of depression and cognitive decline.

In summary, our research establishes an association between impaired lung function and increased depression risk among middle-aged and old people Chinese individuals. These findings underscore the importance of addressing respiratory health as a strategy for depression prevention in this population. Our results indicate that interventions targeting lung function improvement may have significant public health benefits.

## Conclusion

Our findings show that impaired lung function significantly increases depression risk in middle-aged and older adults — a level of risk comparable to well-established psychosocial factors. To put this evidence into action, we propose:

1. Routine PEF screening as part of mental health assessments for aging populations, especially for those with unexplained depressive symptoms.
2. Integrated pulmonary-psychosocial treatments, such as combining breathing retraining with cognitive behavioral therapy, tailored to individuals.
3. Validation of the “pulmonary dysfunction-related depression” concept through mechanistic studies investigating nerve-mediated lung-brain communication.

## The key findings of the research

1. Prevalence of depression: Depression was more prevalent among individuals with poorer lung function compared to those with better lung function.
2. Longitudinal association: Lower peak expiratory flow (PEF) was significantly associated with an increased risk of developing depression over a 3.6-year follow-up period.
3. A linear relationship: A linear relationship was observed between PEF and depression risk, with lower PEF levels associated with higher odds of depression.

## Data Availability

The raw data supporting the conclusions of this article will be made available by the authors, without undue reservation.
