# Supplementary material for: Associations between pulmonary function and depression: evidence from the CHARLS cohort 2015–2018
Source: Front Public Health. 2025 Jul 17;13:1551356. doi: 10.3389/fpubh.2025.1551356 (PMC12310457; doi:10.3389/fpubh.2025.1551356)
Supplement: Supplementary file 1 [file Table_1.docx]

Supplementary Appendix

Table of contents

**Table S1. Baseline characteristics of 15188 participants with depression or not by PEF status in the longitudinal analysis**

**Table S2. Cross-sectional association between PEF status, depression among all participants**

**Table S3. Relative Importance of Variables Based on GBM Analysis**

**Figure S1. GBM model performance evaluation: ROC curve analysis**

**Table S4. Variance Inflation Factor (VIF) Analysis**

**Table S5. Stratified analysis of PEF percentage and depression Stratified by different levels of PEF in Baseline**

**Table S6. Depression Incidence Rates from 2015 to 2018 Stratified by Baseline PEF Percentage Status**

**Table S7-1. Model 1 (Unimputed Data): Basic Adjustment (Age, Gender)**

**TableS 7-2. Model 2 (Unimputed Data): Intermediate Adjustment (Adds Health Status)**

**TableS 7-3. Model 3 (Unimputed Data): Full Adjustment (Adds Socioeconomic & Physical Factors)**

**Table S1.** Baseline characteristics of 15188 participants with depression or not by PEF status in the longitudinal analysis

| **Covariates** | **level** | **Overall** | **Restricted** | **Average** | **Good** | ***p*** |
| --- | --- | --- | --- | --- | --- | --- |
| n |  | 15188 | 4449 | 3837 | 6902 |  |
| **Demographic and Lifestyle** |  |  |  |  |  |  |
| age (mean (SD)) |  | 68.53 (10.08) | 68.50 (10.53) | 66.56 (9.62) | 69.64 (9.87) | <0.001 |
| gender (%) | Male | 7115 (46.85) | 1891 (42.50) | 1668 (43.47) | 3556 (51.52) | <0.001 |
|  | Female | 8073 (53.15) | 2558 (57.50) | 2169 (56.53) | 3346 (48.48) |  |
|  |  |  |  |  |  |  |
| Arm Length (cm) (mean (SD)) |  | 33.56 (11.39) | 33.41 (14.64) | 33.54 (15.83) | 33.67 (2.88) | 0.495 |
| Knee Height (cm) (mean (SD)) |  | 47.82 (13.85) | 47.42 (14.71) | 47.51 (3.65) | 48.25 (16.58) | 0.002 |
| Location (%) | 1 Main City Zone | 1901 (12.56) | 460 (10.37) | 438 (11.45) | 1003 (14.58) | <0.001 |
|  | 2 Combination Zone Between Urban and Rural Areas | 707 (4.67) | 180 (4.06) | 205 (5.36) | 322 (4.68) |  |
|  | 3 The Town Center | 804 (5.31) | 216 (4.87) | 202 (5.28) | 386 (5.61) |  |
|  | 4 ZhenXiang Area | 407 (2.69) | 115 (2.59) | 107 (2.80) | 185 (2.69) |  |
|  | 5 Special Area | 57 (0.38) | 15 (0.34) | 12 (0.31) | 30 (0.44) |  |
|  | 6 Township Central | 211 (1.39) | 73 (1.65) | 45 (1.18) | 93 (1.35) |  |
|  | 7 Village | 11052 (73.00) | 3375 (76.12) | 2816 (73.62) | 4861 (70.65) |  |
| Marital Status (%) | 1 Married with Spouse Present | 12617 (83.07) | 3615 (81.25) | 3228 (84.13) | 5774 (83.66) | <0.001 |
|  | 2 Married But Not Living with Spouse Temporarily for Reasons Such as Work | 725 (4.77) | 227 (5.10) | 201 (5.24) | 297 (4.30) |  |
|  | 3 Separated | 33 (0.22) | 12 (0.27) | 10 (0.26) | 11 (0.16) |  |
|  | 4 Divorced | 123 (0.81) | 42 (0.94) | 26 (0.68) | 55 (0.80) |  |
|  | 5 Widowed | 1581 (10.41) | 510 (11.46) | 344 (8.97) | 727 (10.53) |  |
|  | 6 Never Married | 93 (0.61) | 41 (0.92) | 22 (0.57) | 30 (0.43) |  |
|  | 7 Cohabitated | 16 (0.11) | 2 (0.04) | 6 (0.16) | 8 (0.12) |  |
| Smoking Status (%) | 1 Still Have | 4246 (65.05) | 1225 (64.61) | 1085 (70.50) | 1936 (62.61) | <0.001 |
|  | 2 Quit | 1917 (29.37) | 573 (30.22) | 374 (24.30) | 970 (31.37) |  |
|  | 3 Never Smoked | 364 (5.58) | 98 (5.17) | 80 (5.20) | 186 (6.02) |  |
| Alcohol Use (%) | 1 I Never Had A Drink | 8536 (76.63) | 2692 (78.76) | 2230 (78.36) | 3614 (74.13) | <0.001 |
|  | 2 I Used to Drink Less Than Once A Month | 1282 (11.51) | 332 (9.71) | 308 (10.82) | 642 (13.17) |  |
|  | 3 I Used to Drink More Than Once A Month | 1321 (11.86) | 394 (11.53) | 308 (10.82) | 619 (12.70) |  |
| Days Ag Work (mean (SD)) |  | 5.24 (2.08) | 5.33 (2.03) | 5.26 (2.04) | 5.15 (2.14) | 0.007 |
| Ed Change (%) | 1 No Formal Education (Illiterate) | 2887 (26.43) | 945 (30.11) | 690 (26.34) | 1252 (24.24) | <0.001 |
|  | 2 Did Not Finish Primary School But Capable of Reading/Writing | 2040 (18.68) | 667 (21.26) | 484 (18.47) | 889 (17.22) |  |
|  | 3 Sichu/Home School | 40 (0.37) | 13 (0.41) | 14 (0.53) | 13 (0.25) |  |
|  | 4 Elementary School | 2520 (23.07) | 748 (23.84) | 590 (22.52) | 1182 (22.89) |  |
|  | 5 Middle School | 2292 (20.99) | 553 (17.62) | 593 (22.63) | 1146 (22.19) |  |
|  | 6 High School | 772 (7.07) | 144 (4.59) | 188 (7.18) | 440 (8.52) |  |
|  | 7 Vocational School | 212 (1.94) | 47 (1.50) | 42 (1.60) | 123 (2.38) |  |
|  | 8 Two/Three Year College/Associate Degree | 113 (1.03) | 14 (0.45) | 13 (0.50) | 86 (1.67) |  |
|  | 9 Four-year College/Bachelor's Degree | 45 (0.41) | 7 (0.22) | 6 (0.23) | 32 (0.62) |  |
|  | 10 Master's Degree | 1 (0.01) | 0 (0.00) | 0 (0.00) | 1 (0.02) |  |
| Marriage Sat (%) | 1 Completely Satisfied | 1001 (7.55) | 252 (6.62) | 263 (7.71) | 486 (8.04) | <0.001 |
|  | 2 Very Satisfied | 5574 (42.02) | 1554 (40.82) | 1403 (41.11) | 2617 (43.29) |  |
|  | 3 Some What Satisfied | 5657 (42.65) | 1662 (43.66) | 1456 (42.66) | 2539 (42.00) |  |
|  | 4 Not Very Satisfied | 723 (5.45) | 235 (6.17) | 203 (5.95) | 285 (4.71) |  |
|  | 5 Not at All Satisfied | 305 (2.30) | 102 (2.68) | 88 (2.58) | 115 (1.90) |  |
|  | 6 No Spouse Now | 5 (0.04) | 2 (0.05) | 0 (0.00) | 3 (0.05) |  |
| Mos Ag Work (mean (SD)) |  | 6.40 (4.17) | 6.68 (4.17) | 6.36 (4.16) | 6.23 (4.18) | <0.001 |
| Childhood Health (%) | 1 Excellent | 1738 (11.59) | 440 (10.03) | 431 (11.38) | 867 (12.70) | <0.001 |
|  | 2 Very Good | 6068 (40.46) | 1617 (36.88) | 1521 (40.17) | 2930 (42.93) |  |
|  | 3 Good | 2847 (18.99) | 911 (20.78) | 720 (19.02) | 1216 (17.82) |  |
|  | 4 Fair | 3372 (22.49) | 1085 (24.74) | 877 (23.16) | 1410 (20.66) |  |
|  | 5 Poor | 971 (6.48) | 332 (7.57) | 237 (6.26) | 402 (5.89) |  |
| Midday Sleep Time (hrs) (mean (SD)) | | 38.75 (44.95) | 38.55 (45.55) | 37.25 (44.94) | 39.71 (44.55) | 0.024 |
| Sleep Time (hrs) (mean (SD)) |  | 6.40 (1.92) | 6.37 (2.03) | 6.41 (1.92) | 6.42 (1.85) | 0.333 |
| **Self-Reported Health** |  |  |  |  |  |  |
| Health Sat (%) | 1 Completely Satisfied | 544 (3.61) | 140 (3.18) | 144 (3.78) | 260 (3.79) | <0.001 |
|  | 2 Very Satisfied | 3190 (21.16) | 894 (20.30) | 777 (20.39) | 1519 (22.15) |  |
|  | 3 Some What Satisfied | 7442 (49.37) | 2038 (46.27) | 1865 (48.94) | 3539 (51.60) |  |
|  | 4 Not Very Satisfied | 2937 (19.48) | 960 (21.79) | 769 (20.18) | 1208 (17.61) |  |
|  | 5 Not at All Satisfied | 961 (6.38) | 373 (8.47) | 256 (6.72) | 332 (4.84) |  |
| Toilet Diff (%) | 1 No, I Don't Have Any Difficulty | 8239 (81.57) | 2623 (80.14) | 2040 (81.89) | 3576 (82.47) | 0.044 |
|  | 2 I Have Difficulty But Can Still Do It | 1564 (15.49) | 532 (16.25) | 390 (15.66) | 642 (14.81) |  |
|  | 3 Yes, I Have Difficulty and Need Help | 167 (1.65) | 71 (2.17) | 33 (1.32) | 63 (1.45) |  |
|  | 4 I Can Not Do It | 130 (1.29) | 47 (1.44) | 28 (1.12) | 55 (1.27) |  |
| Life Sat (%) | 1 Completely Satisfied | 995 (6.59) | 281 (6.37) | 236 (6.18) | 478 (6.95) | 0.001 |
|  | 2 Very Satisfied | 5510 (36.49) | 1608 (36.48) | 1362 (35.69) | 2540 (36.95) |  |
|  | 3 Some What Satisfied | 7326 (48.52) | 2091 (47.44) | 1880 (49.27) | 3355 (48.81) |  |
|  | 4 Not Very Satisfied | 1015 (6.72) | 335 (7.60) | 273 (7.15) | 407 (5.92) |  |
|  | 5 Not at All Satisfied | 252 (1.67) | 93 (2.11) | 65 (1.70) | 94 (1.37) |  |
| Self rated memory (%) | 1 Excellent | 127 (0.84) | 31 (0.70) | 34 (0.89) | 62 (0.90) | <0.001 |
|  | 2 Very good | 835 (5.50) | 228 (5.13) | 211 (5.50) | 396 (5.74) |  |
|  | 3 Good | 1142 (7.53) | 307 (6.91) | 290 (7.57) | 545 (7.90) |  |
|  | 4 Fair | 8118 (53.51) | 2264 (50.97) | 2021 (52.73) | 3833 (55.58) |  |
|  | 5 Poor | 4949 (32.62) | 1612 (36.29) | 1277 (33.32) | 2060 (29.87) |  |
| Sitting Diff (%) | 1 No, I Don't Have Any Difficulty | 10532 (71.63) | 2943 (68.11) | 2643 (72.04) | 4946 (73.68) | <0.001 |
|  | 2 I Have Difficulty But Can Still Do It | 3842 (26.13) | 1241 (28.72) | 952 (25.95) | 1649 (24.56) |  |
|  | 3 Yes, I Have Difficulty and Need Help | 210 (1.43) | 84 (1.94) | 54 (1.47) | 72 (1.07) |  |
|  | 4 I Can Not Do It | 119 (0.81) | 53 (1.23) | 20 (0.55) | 46 (0.69) |  |
| Stooping Diff (%) | 1 No, I Don't Have Any Difficulty | 9945 (67.70) | 2719 (62.98) | 2513 (68.53) | 4713 (70.29) | <0.001 |
|  | 2 I Have Difficulty But Can Still Do It | 3208 (21.84) | 997 (23.09) | 778 (21.22) | 1433 (21.37) |  |
|  | 3 Yes, I Have Difficulty and Need Help | 270 (1.84) | 104 (2.41) | 73 (1.99) | 93 (1.39) |  |
|  | 4 I Can Not Do It | 1266 (8.62) | 497 (11.51) | 303 (8.26) | 466 (6.95) |  |
| Difficulty with Climbing (%) | 1 No, I Don't Have Any Difficulty | 8563 (58.79) | 2185 (51.18) | 2150 (59.24) | 4228 (63.42) | <0.001 |
|  | 2 I Have Difficulty But Can Still Do It | 4021 (27.61) | 1262 (29.56) | 1004 (27.67) | 1755 (26.32) |  |
|  | 3 Yes, I Have Difficulty and Need Help | 348 (2.39) | 133 (3.12) | 84 (2.31) | 131 (1.96) |  |
|  | 4 I Can Not Do It | 1633 (11.21) | 689 (16.14) | 391 (10.77) | 553 (8.29) |  |
| Health Change (%) | 1 Better | 1442 (9.68) | 467 (10.71) | 342 (9.11) | 633 (9.33) | <0.001 |
|  | 2 About The Same | 7120 (47.79) | 1886 (43.25) | 1820 (48.47) | 3414 (50.34) |  |
|  | 3 Worse | 6336 (42.53) | 2008 (46.04) | 1593 (42.42) | 2735 (40.33) |  |
| Disease Aware (%) | 1 Yes | 105 (0.71) | 35 (0.81) | 29 (0.77) | 41 (0.61) | <0.001 |
|  | 2 No | 12960 (87.18) | 3657 (84.40) | 3281 (87.24) | 6022 (88.92) |  |
|  | 3 Don't Know | 1801 (12.11) | 641 (14.79) | 451 (11.99) | 709 (10.47) |  |
| Self-rated Health (%) | 1 Excellent | 117 (1.52) | 26 (1.15) | 33 (1.70) | 58 (1.67) | <0.001 |
|  | 2 Very Good | 947 (12.32) | 224 (9.88) | 237 (12.20) | 486 (13.98) |  |
|  | 3 Good | 955 (12.43) | 239 (10.54) | 235 (12.09) | 481 (13.84) |  |
|  | 4 Fair | 4349 (56.58) | 1267 (55.89) | 1105 (56.87) | 1977 (56.88) |  |
|  | 5 Poor | 1318 (17.15) | 511 (22.54) | 333 (17.14) | 474 (13.64) |  |
| Ill in Last Month (%) | 1 Yes | 1814 (14.30) | 609 (16.78) | 473 (14.78) | 732 (12.51) | <0.001 |
|  | 2 No | 10867 (85.70) | 3020 (83.22) | 2727 (85.22) | 5120 (87.49) |  |
| Disability (%) | 1 Yes | 2181 (14.38) | 812 (18.30) | 547 (14.27) | 822 (11.92) | <0.001 |
|  | 2 No | 12014 (79.20) | 3283 (73.97) | 3089 (80.59) | 5642 (81.79) |  |
|  | 3 Too Old to Do The Work | 974 (6.42) | 343 (7.73) | 197 (5.14) | 434 (6.29) |  |
| Lifting Diff (%) | 1 No, I Don't Have Any Difficulty | 12794 (87.16) | 3528 (81.82) | 3190 (87.13) | 6076 (90.62) | <0.001 |
|  | 2 I Have Difficulty But Can Still Do It | 744 (5.07) | 267 (6.19) | 215 (5.87) | 262 (3.91) |  |
|  | 3 Yes, I Have Difficulty and Need Help | 145 (0.99) | 55 (1.28) | 40 (1.09) | 50 (0.75) |  |
|  | 4 I Can Not Do It | 995 (6.78) | 462 (10.71) | 216 (5.90) | 317 (4.73) |  |
| Local Service Sat (%) | 1 Very Satisfied | 2299 (15.64) | 722 (16.78) | 582 (15.62) | 995 (14.92) | 0.023 |
|  | 2 Somewhat Satisfied | 3564 (24.25) | 1041 (24.20) | 870 (23.35) | 1653 (24.78) |  |
|  | 3 Neutral | 6001 (40.83) | 1758 (40.86) | 1528 (41.01) | 2715 (40.70) |  |
|  | 4 Somewhat disSatisfied | 1809 (12.31) | 472 (10.97) | 479 (12.86) | 858 (12.86) |  |
|  | 5 Very disSatisfied | 1025 (6.97) | 309 (7.18) | 267 (7.17) | 449 (6.73) |  |
| Run/Jog 1 Km (%) | 1 No, I Don't Have Any Difficulty | 7011 (48.89) | 1675 (39.82) | 1778 (49.79) | 3558 (54.20) | <0.001 |
|  | 2 I Have Difficulty But Can Still Do It | 1587 (11.07) | 440 (10.46) | 417 (11.68) | 730 (11.12) |  |
|  | 3 Yes, I Have Difficulty and Need Help | 189 (1.32) | 63 (1.50) | 51 (1.43) | 75 (1.14) |  |
|  | 4 I Can Not Do It | 5554 (38.73) | 2028 (48.22) | 1325 (37.10) | 2201 (33.53) |  |
| Vision status (mean (SD)) |  | 0.69 (0.46) | 0.72 (0.45) | 0.70 (0.46) | 0.68 (0.47) | <0.001 |
| Auditory status (mean (SD)) |  | 0.65 (0.48) | 0.67 (0.47) | 0.65 (0.48) | 0.63 (0.48) | <0.001 |
| **Label index** |  |  |  |  |  |  |
| Systolic BP, mmHg (mean (SD)) |  | 129.19 (46.45) | 128.41 (44.58) | 127.96 (42.22) | 130.37 (49.73) | 0.016 |
| Pulse, bpm (mean (SD)) |  | 74.20 (10.83) | 75.01 (11.35) | 74.05 (10.61) | 73.76 (10.57) | <0.001 |
| Platelets, x10^9/L (mean (SD)) |  | 204.93 (74.73) | 207.13 (75.32) | 203.06 (73.00) | 204.58 (75.27) | 0.079 |
| Diastolic BP, mmHg (mean (SD)) |  | 75.19 (12.14) | 74.76 (12.57) | 75.32 (12.16) | 75.39 (11.84) | 0.02 |
| maxPEF, L/min (mean (SD)) |  | 321.66 (125.39) | 198.57 (72.28) | 309.06 (71.93) | 408.02 (105.07) | <0.001 |
| HDL Chol, mg/dL (mean (SD)) |  | 51.22 (11.50) | 51.77 (11.65) | 51.19 (11.47) | 50.90 (11.42) | 0.002 |
| Hemoglobin, g/dL (mean (SD)) |  | 13.72 (1.95) | 13.60 (1.97) | 13.68 (1.99) | 13.81 (1.90) | <0.001 |
| CRP, mg/L(mean (SD)) |  | 2.65 (5.63) | 2.96 (6.07) | 2.63 (5.77) | 2.48 (5.24) | <0.001 |
| WBC, x10^9/L(mean (SD)) |  | 5.99 (2.07) | 6.03 (1.88) | 5.96 (2.67) | 5.99 (1.79) | 0.393 |
| HbA1c , (%) (mean (SD)) |  | 5.97 (0.98) | 5.95 (0.94) | 5.93 (0.97) | 6.00 (1.02) | 0.002 |
| Total Chol, mg/dL (mean (SD)) |  | 183.91 (36.37) | 181.70 (36.86) | 183.60 (36.17) | 185.46 (36.10) | <0.001 |
| **Economic Indicators** |  |  |  |  |  |  |
| New Rural Pension (%) | 1 Participate | 4672 (54.95) | 1436 (54.44) | 1373 (62.21) | 1863 (50.93) | <0.001 |
|  | 2 Receive | 3831 (45.05) | 1202 (45.56) | 834 (37.79) | 1795 (49.07) |  |
| Hrs Ag Work (mean (SD)) |  | 6.45 (2.99) | 6.62 (2.92) | 6.50 (2.97) | 6.30 (3.04) | 0.001 |
|  |  |  |  |  |  |  |
| Pension Insur (%) | 1 Yes, Have Quali?cation | 8214 (56.43) | 2464 (58.04) | 2129 (57.79) | 3621 (54.63) | <0.001 |
|  | 2 Yes, Other Reason(fn | 727 (4.99) | 227 (5.35) | 187 (5.08) | 313 (4.72) |  |
|  | 3 No, Participate Government or Firm Pension | 1520 (10.44) | 300 (7.07) | 318 (8.63) | 902 (13.61) |  |
|  | 4 No, Local Community Not Execute | 1952 (13.41) | 576 (13.57) | 502 (13.63) | 874 (13.19) |  |
|  | 5 No, Other Reason(fn | 2144 (14.73) | 678 (15.97) | 548 (14.88) | 918 (13.85) |  |
| **Composite indicators** |  |  |  |  |  |  |
| TyG_BMI (mean (SD)) |  | 32.65 (3.91) | 32.17 (4.10) | 32.62 (3.90) | 32.97 (3.77) | <0.001 |
| ASM/Ht^2 score (mean (SD)) |  | 11.83 (3.14) | 11.12 (3.22) | 11.83 (3.10) | 12.29 (3.01) | <0.001 |
| Sarcopenia (%) | No | 14167 (94.57) | 3984 (91.10) | 3590 (94.87) | 6593 (96.61) | <0.001 |
|  | Yes | 814 (5.43) | 389 (8.90) | 194 (5.13) | 231 (3.39) |  |
| rec_score (mean (SD)) |  | 13.12 (5.77) | 11.97 (5.83) | 13.17 (5.72) | 13.83 (5.65) | <0.001 |
| IC_score (mean (SD)) |  | 4.27 (1.38) | 4.49 (1.35) | 4.27 (1.37) | 4.13 (1.38) | <0.001 |
| rec_score_level (%) | Good | 2765 (18.21) | 577 (12.97) | 690 (17.98) | 1498 (21.70) | <0.001 |
|  | Bad | 12423 (81.79) | 3872 (87.03) | 3147 (82.02) | 5404 (78.30) |  |
| PEF Percentage (mean (SD)) |  | 96.52 (31.88) | 60.10 (15.85) | 90.22 (5.66) | 123.51 (21.24) | <0.001 |
| Depression (%) | Normal | 9921 (65.32) | 2619 (58.87) | 2487 (64.82) | 4815 (69.76) | <0.001 |
|  | Mild | 2638 (17.37) | 839 (18.86) | 675 (17.59) | 1124 (16.29) |  |
|  | Moderate | 1585 (10.44) | 567 (12.74) | 402 (10.48) | 616 (8.92) |  |
|  | Severe | 1044 (6.87) | 424 (9.53) | 273 (7.11) | 347 (5.03) |  |

**Table S2**. Cross-sectional association between PEF status, depression among all participants

| **Covariates** | level | Overall | **Restricted** | **Average** | **Good** | p |
| --- | --- | --- | --- | --- | --- | --- |
| n |  | 12304 | 3481 | 3143 | 5680 |  |
| age (mean (SD)) |  | 67.89 (9.43) | 67.57 (9.80) | 65.98 (8.98) | 69.15 (9.26) | <0.001 |
| maxPEF (mean (SD)) |  | 327.29 (123.08) | 203.77 (69.59) | 311.76 (69.14) | 411.57 (103.05) | <0.001 |
| TyG_BMI (mean (SD)) |  | 32.74 (3.91) | 32.32 (4.09) | 32.67 (3.91) | 33.03 (3.77) | <0.001 |
| rec_score (mean (SD)) |  | 11.41 (5.55) | 10.58 (5.56) | 11.50 (5.58) | 11.86 (5.47) | <0.001 |
| rec_score_level (%) | Good | 986 (8.01) | 218 (6.26) | 256 (8.15) | 512 (9.01) | <0.001 |
|  | Bad | 11318 (91.99) | 3263 (93.74) | 2887 (91.85) | 5168 (90.99) |  |
| depression (%) | Normal | 7420 (60.31) | 1862 (53.49) | 1853 (58.96) | 3705 (65.23) | <0.001 |
|  | Mild | 2259 (18.36) | 669 (19.22) | 601 (19.12) | 989 (17.41) |  |
|  | Moderate | 1504 (12.22) | 532 (15.28) | 388 (12.34) | 584 (10.28) |  |
|  | Severe | 1121 (9.11) | 418 (12.01) | 301 (9.58) | 402 (7.08) |  |
| IC_score (mean (SD)) |  | 4.92 (1.20) | 5.06 (1.17) | 4.92 (1.21) | 4.82 (1.19) | <0.001 |
| ASM/Ht^2 score (mean (SD)) | | 11.97 (3.08) | 11.30 (3.14) | 11.93 (3.04) | 12.40 (2.99) | <0.001 |
| Sarcopenia (%) | No | 11598 (95.39) | 3171 (92.58) | 2973 (95.63) | 5454 (96.96) | <0.001 |
|  | Yes | 561 (4.61) | 254 (7.42) | 136 (4.37) | 171 (3.04) |  |

**Table S3**. Relative Importance of Variables Based on GBM Analysis

| **Type** | **Var_describe** | **Standard Histogram Label** | **rel.inf** |
| --- | --- | --- | --- |
| Self-Reported Health | How Satisfied Are You with Your Health? | Satisfaction with Health (Very Satisfied, Satisfied, Neither Satisfied nor Dissatisfied, Dissatisfied, or Very Dissatisfied) | 12.28436 |
| Self-Reported Health | Difficulty with Using The Toilet | Difficulty with Using the Toilet (Never, Occasionally, Sometimes, Often, or Always) | 8.842078 |
| Self-Reported Health | Life Satisfaction | Life Satisfaction (Very Satisfied, Satisfied, Neither Satisfied nor Dissatisfied, Dissatisfied, or Very Dissatisfied) | 7.817504 |
| Self-Reported Health | Self rated memory | Self-rated Memory (Excellent, Good, Fair, or Poor) | 6.577739 |
| Self-Reported Health | Sleeping Time | Sleeping Time (hours) | 5.968625 |
| Self-Reported Health | rec_score | REC score | 4.948275 |
| Self-Reported Health | Difficulty with Sitting | Difficulty with Sitting (Never, Occasionally, Sometimes, Often, or Always) | 3.97603 |
| Self-Reported Health | How Satisfied Are You with Your Marriage (Relationship with | Satisfaction with Marriage (Very Satisfied, Satisfied, Neither Satisfied nor Dissatisfied, Dissatisfied, or Very Dissatisfied) | 3.918486 |
| Self-Reported Health | Difficulty with Stooping, Kneeling, Crouching | Difficulty with Stooping, Kneeling, Crouching (Never, Occasionally, Sometimes, Often, or Always) | 2.928721 |
| Self-Reported Health | db005_y | db005_y | 2.86995 |
| Self-Reported Health | Compared Health Status with Last Interview | Change in Health Status (Improved, Worsened, or Stayed the Same) | 2.732436 |
| Self-Reported Health | Self Aware of Disease | Self-awareness of Disease (Yes or No) | 2.703216 |
| demographic and lifestyle | Age | Age (years) | 1.929138 |
| Self-Reported Health | Self Comment of Your Health | Self-rated Health (Excellent, Good, Fair, or Poor) | 1.707177 |
| Self-Reported Health | Have You Been Ill in the Last Month? | Illness in the Last Month (Yes or No) | 1.614647 |
| label | Systolic Reading | Systolic Blood Pressure (mmHg) | 1.522689 |
| Self-Reported Health | Can’t Do Specific Work For Long Time Because of Disabilities | Disability (Yes or No) | 1.471865 |
| demographic and lifestyle | Hours for Household Agricultural Work | Hours of Household Agricultural Work | 1.452881 |
| Self-Reported Health | Difficulty with Lifting | Difficulty with Lifting (Never, Occasionally, Sometimes, Often, or Always) | 1.398156 |
| label | PULSE | Pulse Rate (beats per minute) | 1.299614 |
| label | Platelets (109/L) | Platelet Count (10^9/L) | 1.296308 |
| demographic and lifestyle | Arm Length | Arm Length (cm) | 1.214715 |
| label | Total Cholesterol (mg/dl) | Total Cholesterol (mg/dL) | 1.210874 |
| Self-Reported Health | Your Health During Childhood | Childhood Health (Excellent, Good, Fair, or Poor) | 1.184387 |
| label | Diastolic Reading | Diastolic Blood Pressure (mmHg) | 1.178601 |
| label | ASM/Ht^2 | ASM/Ht^2 score | 1.030029 |
| demographic and lifestyle | Was It Village or City/Town | Location (Village or City/Town) | 1.016853 |
| label | maxPEF | Peak Expiratory Flow (L/s) | 0.930547 |
| Self-Reported Health | Satisfaction with Quality, Cost, and Convenience of Local | Satisfaction with Local Health Services (Very Satisfied, Satisfied, Neither Satisfied nor Dissatisfied, Dissatisfied, or Very Dissatisfied) | 0.91862 |
| Self-Reported Health | eyesight | Eyesight (Good, Fair, or Poor) | 0.873314 |
| label | TyG_BMI | TyG-BMI | 0.853257 |
| Self-Reported Health | hearing | Hearing (Good, Fair, or Poor) | 0.815604 |
| Self-Reported Health | Have Qualification to Participate Residents’ Pension Insur- | Participation in Residents' Pension Insurance (Yes or No) | 0.815479 |
| demographic and lifestyle | Martial Status | Marital Status | 0.804741 |
| label | HDL Cholesterol (mg/dl) | HDL Cholesterol (mg/dL) | 0.769891 |
| demographic and lifestyle | Days for Household Agricultural Work | Days of Household Agricultural Work | 0.668631 |
| label | Hemoglobin (g/dl) | Hemoglobin (g/dL) | 0.625412 |
| Self-Reported Health | Sleeping Time | Sleeping Time (hours) | 0.605069 |
| demographic and lifestyle | Gender | Gender (Male or Female) | 0.599919 |
| demographic and lifestyle | Have Your Highest Level of Education Changed? | Change in Education Level (Yes or No) | 0.577118 |
| demographic and lifestyle | Months for Household Agricultural Work | Months of Household Agricultural Work | 0.555901 |
| demographic and lifestyle | Knee Height | Knee Height (cm) | 0.525152 |
| label | C-Reactive Protein (CRP) (mg/l) | C-reactive Protein (mg/L) | 0.51446 |
| Self-Reported Health | Eyesight for Seeing Things up Close | Near Vision (Good, Fair, or Poor) | 0.505192 |
| label | White Blood Cell (in thousands) | White Blood Cell Count (10^3/?L) | 0.455729 |
| Self-Reported Health | Running or Jogging about 1 Km | Ability to Run or Jog 1 Km (Yes or No) | 0.416987 |
| label | Glycated Hemoglobin (%) | Glycated Hemoglobin (%) | 0.31725 |
| Self-Reported Health | Hearing Problem | Hearing Problem (Yes or No) | 0.213616 |
| demographic and lifestyle | Still Smoke or Quit | Smoking Status (Current Smoker, Former Smoker, or Never Smoker) | 0.207252 |
| demographic and lifestyle | Ever Drink Alcoholic Beverages | Alcohol Consumption (Yes or No) | 0.163965 |
| label | Peak expiratory flow | Group 1,2,3 | 0.080312 |
| Self-Reported Health | You Are Currently Participating or Receiving New Rural | Participation in New Rural Pension Scheme (Yes or No) | 0.046311 |
| label | Sarcopenia | Sarcopenia (Yes or No) | 0.044921 |

**Figure S1.** GBM model performance evaluation: ROC curve analysis


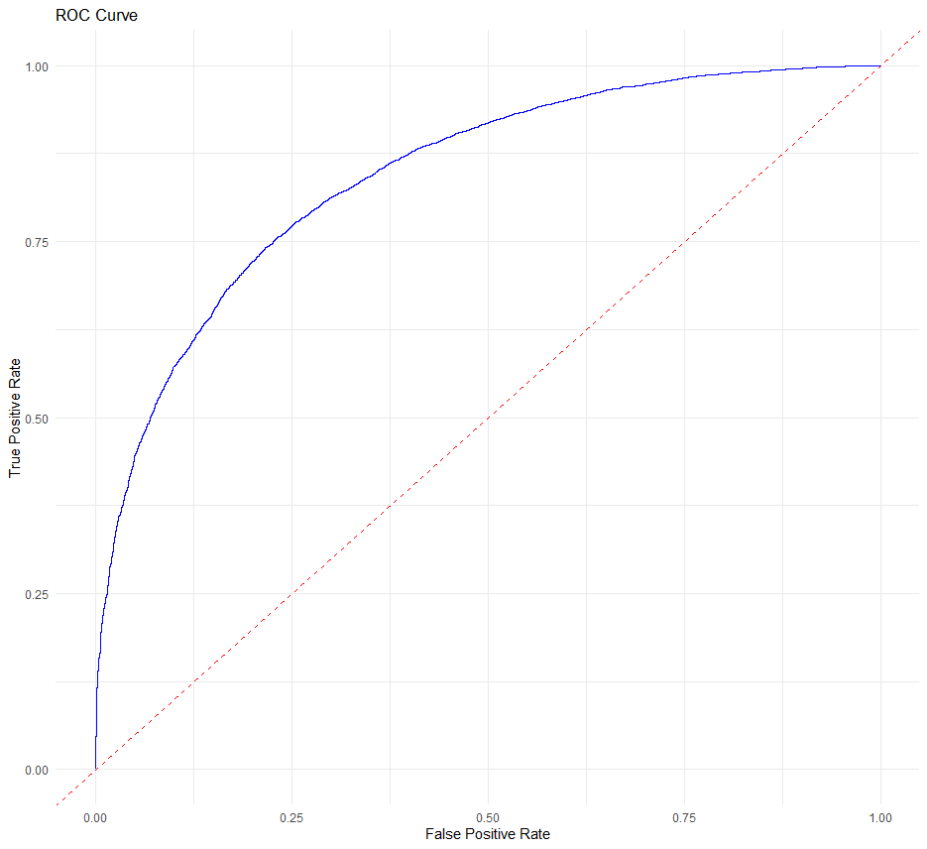


**Table S4.** Variance Inflation Factor (VIF) Analysis

|  | **GVIF** | **Df** | **GVIF^(1/(2*Df))** |
| --- | --- | --- | --- |
| Group | 1.068784 | 2 | 1.016769 |
| age | 1.428362 | 1 | 1.195141 |
| gender | 1.503552 | 1 | 1.226194 |
| dc042_w3 | 1.389753 | 4 | 1.041999 |
| da048 | 1.120061 | 4 | 1.014274 |
| dc043_w3 | 1.24348 | 5 | 1.022031 |
| ASMHI2 | 1.573762 | 1 | 1.254497 |
| qa007 | 1.082321 | 1 | 1.040347 |
| qa013 | 1.015068 | 1 | 1.007506 |
| eyesight | 1.170928 | 1 | 1.082094 |
| hearing | 1.205759 | 1 | 1.098071 |
| eh007_w3 | 1.197482 | 4 | 1.022783 |
| fn069_w3 | 1.056936 | 4 | 1.006946 |
| dc004 | 1.191207 | 4 | 1.022112 |
| da050 | 1.021152 | 1 | 1.010521 |
| db006 | 1.38351 | 3 | 1.055594 |
| db004 | 1.345251 | 3 | 1.050672 |

**Table S5:** Stratified analysis of PEF percentage and depression Stratified by different levels of PEF in Baseline

| **PEF Percentage** | **Restricted** | **Average** | **Good** | **P value** | **P for trend** | **Per 1 SD increase** |
| --- | --- | --- | --- | --- | --- | --- |
| Median（PEF Percentage） | 60.10 (15.85) | 90.22 (5.66) | 123.51 (21.24) |  | - | - |
| Cases, n (%) | 4449 | 3837 | 6902 |  | - | - |
| Crude, OR (95% CI) | reference | 0.777(0.711-0.849) | 0.620(0.573-0.671) | <0.001 | < 0.001 | 0.802(0.775-0.83) |
| Model 1, OR (95% CI) | reference | 0.804(0.734-0.880) | 0.635(0.586-0.688) | <0.001 | < 0.001 | 0.804(0.777-0.833) |
| Model 2, OR (95% CI) | reference | 0.845(0.767-0.931) | 0.714(0.656-0.778) | 0.006 | < 0.001 | 0.846(0.815-0.878) |
| Model 3, OR (95% CI) | reference | 0.864(0.779-0.959) | 0.812(0.740-0.891) | <0.001 | < 0.001 | 0.898(0.862-0.935) |

**Table S6:** Depression Incidence Rates from 2015 to 2018 Stratified by Baseline PEF Percentage Status

| PEF_Percentage | New Cases of depression(%n) | Incidence Rate, per 1000 | **Model 1**a | **Model 2**b | **Model 3**c |
| --- | --- | --- | --- | --- | --- |
| **Depression** |  |  |  |  |  |
| Restricted | 90 | 7.60 | Reference | Reference | Reference |
| Average | 86 | 8.05 | 0.817 (0.740 - 0.902)*** | 0.839 (0.757 - 0.930)*** | 0.888 (0.789 - 1.000) |
| Good | 130 | 6.73 | 0.633 (0.579 - 0.690)*** | 0.681 (0.622 - 0.746)*** | 0.776 (0.698 - 0.864)*** |
| **Cognitive Dysfunction** |  |  |  |  |  |
| Restricted | 257 | 21.71 | Reference | Reference | Reference |
| Average | 326 | 30.51 | 0.900 (0.790 - 1.026) | 0.892 (0.781 - 1.018) | 0.773 (0.672 - 0.890)*** |
| Good | 781 | 40.44 | 0.788 (0.702 - 0.885)*** | 0.796 (0.707 - 0.895)*** | 0.633 (0.558 - 0.717)*** |

**Table S7-1.** Model 1 (Unimputed Data): Basic Adjustment (Age, Gender)

Table S7-1,2,3 reveals a robust inverse relationship between higher PEF groups and depression risk, persisting across increasingly complex statistical models. While the unimputed data provided strong evidence linking pulmonary function to mental health outcomes, future studies should employ more advanced techniques for handling missing data. Nevertheless, our current analysis clearly demonstrates that better pulmonary function may help protect against depression.

| **Variable** | **OR (95% CI)** |
| --- | --- |
| Intercept | 0.13 (0.10–0.17) |
| **PEF Groups (Ref: Average PEF)** |  |
| Good PEF | 0.80 (0.73–0.88) |
| Restricted PEF | 0.63 (0.59–0.69) |
| Age (per year) | 1.02 (1.02–1.02) |
| **Gender (Ref: Male)** |  |
| Female | 1.94 (1.81–2.08) |

**TableS 7-2.** Model 2 (Unimputed Data): Intermediate Adjustment (Adds Health Status)

| **Variable** | **OR (95% CI)** |
| --- | --- |
| Intercept | 0.05 (0.03–0.07) |
| **PEF Groups (Ref: Average PEF)** |  |
| Good PEF | 0.84 (0.77–0.93) |
| Restricted PEF | 0.72 (0.66–0.78) |
| Age (per year) | 1.02 (1.02–1.02) |
| **Gender (Ref: Male)** |  |
| Female | 1.86 (1.72–2.00) |
| **Self-reported Health (Ref: Very Satisfied)** |  |
| Somewhat Satisfied | 1.54 (1.23–1.94) |
| Not Very Satisfied | 4.90 (3.90–6.22) |
| Not at All Satisfied | 14.49 (11.10–19.08) |
| **Childhood Health (Ref: Very Good)** |  |
| Good | 1.21 (1.05–1.39) |
| Fair | 1.41 (1.24–1.62) |
| Poor | 1.76 (1.47–2.10) |

**TableS 7-3.** Model 3 (Unimputed Data): Full Adjustment (Adds Socioeconomic & Physical Factors)

| Variable | OR (95% CI) |
| --- | --- |
| Intercept | 0.21 (0.08–0.52) |
| **PEF Groups (Ref: Average PEF)** |  |
| Good PEF | 0.87 (0.77–0.98) |
| Restricted PEF | 0.81 (0.73–0.91) |
| **Age (per year)** | 0.99 (0.99–1.00) |
| **Gender (Ref: Male)** |  |
| Female | 1.20 (1.08–1.34) |
| **Self-reported Health (Ref: Very Satisfied)** |  |
| Somewhat Satisfied | 1.36 (1.00–1.86) |
| Not Very Satisfied | 2.84 (2.08–3.91) |
| Not at All Satisfied | 5.78 (4.06–8.31) |
| Childhood Health (Ref: Very Good) |  |
| Good | 0.95 (0.81–1.11) |
| Fair | 1.18 (1.00–1.40) |
| Poor | 1.50 (1.20–1.87) |
| **Psychosocial & Economic Factors** |  |
| Satisfaction with Marriage (Ref: Very Satisfied)† | – |
| Somewhat Satisfied | 1.26 (1.03–1.54) |
| Not Very Satisfied | 1.58 (1.29–1.94) |
| Not at All Satisfied | 3.66 (2.80–4.81) |
| No Spouse | 4.27 (2.97–6.20) |
| Pension Insurance (Ref: Yes) | 0.55 (0.46–0.66) |
| Physical Function |  |
| Eyesight Impairment (Ref: Normal) | 1.39 (1.24–1.56) |
| Hearing Impairment (Ref: Normal) | 1.32 (1.18–1.47) |
| **Difficulty Stooping/Kneeling (Ref: None)** | – |
| Mild | 1.69 (1.50–1.89) |
| Moderate | 2.24 (1.60–3.15) |
| Severe | 1.80 (1.51–2.15) |

†Marital dissatisfaction categories (Somewhat/Not Very/Not at All Satisfied) showed graded risk.
